# Spatial mapping of the collagen distribution in human and mouse tissues by force volume atomic force microscopy

**DOI:** 10.1038/s41598-020-72564-9

**Published:** 2020-09-24

**Authors:** Annalisa Calò, Yevgeniy Romin, Rami Srouji, Constantinos P. Zambirinis, Ning Fan, Anthony Santella, Elvin Feng, Sho Fujisawa, Mesruh Turkekul, Sharon Huang, Amber L. Simpson, Michael D’Angelica, William R. Jarnagin, Katia Manova-Todorova

**Affiliations:** 1 Molecular Cytology Core Facility, Memorial Sloan Kettering Cancer Center, 417E 68th St, New York, NY 10065 USA; 2 Physics Faculty, University of Barcelona (UB), Carrer de Martí i Franquès 1-11, 08028 Barcelona, Spain; 3 Hepatopancreatobiliary Service, Department of Surgery, Memorial Sloan Kettering Cancer Center, 1275 York Ave, New York, NY 10065 USA; 4 Washington University School of Medicine, 660 S. Euclid Ave, St. Louis, MO 63110 USA; 5 Department of Surgery, Warren Alpert Medical School of Brown University, 593 Eddy street, APC-4, Providence, RI 02903 USA; 6 School of Computing, Queen’s University, 557 Goodwin Hall, Kingston, ON K7L 2N8 Canada

**Keywords:** Biophysics, Medical research, Engineering

## Abstract

Changes in the elastic properties of living tissues during normal development and in pathological processes are often due to modifications of the collagen component of the extracellular matrix at various length scales. Force volume AFM can precisely capture the mechanical properties of biological samples with force sensitivity and spatial resolution. The integration of AFM data with data of the molecular composition contributes to understanding the interplay between tissue biochemistry, organization and function. The detection of micrometer-size, heterogeneous domains at different elastic moduli in tissue sections by AFM has remained elusive so far, due to the lack of correlations with histological, optical and biochemical assessments. In this work, force volume AFM is used to identify collagen-enriched domains, naturally present in human and mouse tissues, by their elastic modulus. Collagen identification is obtained in a robust way and affordable timescales, through an optimal design of the sample preparation method and AFM parameters for faster scan with micrometer resolution. The choice of a separate reference sample stained for collagen allows correlating elastic modulus with collagen amount and position with high statistical significance. The proposed preparation method ensures safe handling of the tissue sections guarantees the preservation of their micromechanical characteristics over time and makes it much easier to perform correlation experiments with different biomarkers independently.

## Introduction

Physiological and pathological modifications in the composition of the extracellular matrix critically influence the mechanical properties of living tissues, especially when components with support function are involved (e.g. collagen, proteoglycans, laminin)^1,2^. Micro- and nano-mechanical information^3–5^ has been used to address important biological questions from mechanosensing^6–10^, to finding cancer fingerprints^4,11,12^, targeting metastases^13^, and unveiling the mechanism of mechanical failure in connective tissues^5,14^. When used in force volume mode^3,15^, the atomic force microscope provides mechanical information as elastic modulus maps in a quantitative manner, with lateral resolution down to the nanometer (nm) and force sensitivity in piconewtons (pN)^16^. The detection of mechanical forces between the probe and the sample by AFM has enabled a deeper characterization of biological samples. Mechanobiology, in fact, helps to understand how physical forces and mechanical properties affect the function of the molecules, organelles, cells and tissues^17^. In AFM it is possible, by selecting the probe spring constant and tip diameter, to define the relevant scales of the sample inspection. Nanometer-sized probes, with typical diameters below 10 nm, allow exploring sub-micrometer regions of the sample. These probes have been implemented in dedicated setups for high-speed and high-resolution imaging to detect protein motion and study dynamic processes in biological membranes with spatiotemporal resolution (~ 1 nm lateral, ~ 0.1 vertical, 100 ms temporal resolution)^18–20^. The force volume AFM using micrometer-sized probes (beads, microspheres) does not provide such nanoscale resolution, due to the probe’s size. Nevertheless, it has a great potential for screening large areas of biological samples with timescales compatible with many biological processes^21^. The implementation of force volume AFM using micrometer-sized probes for the routine mapping of tissues elastic modulus needs methodical optimizations, in order to overcome the intrinsic slowness of the technique (in AFM, around ten minutes are necessary to collect a single image using a conventional microscope configuration)^3,4,16^, adjust resolution and establish criteria to select regions of interest (ROIs) appropriately. Furthermore, the sample preparation method needs to be properly designed, to avoid imaging artefacts and ensure conditions as close as possible to native ones^17^. The use of force volume AFM with micrometer-sized probes is an optimal choice for studies in biology, when the areas of interest are larger than the probe size and smaller than the maximum x–y piezo extension (around 100 μm for most of the commercial AFMs)^22^. A large variety of tissues exhibit domains of different biochemical composition, which span areas from tens to hundreds of micrometers in the lateral size^4,14,23,24^.

In this work, force volume AFM is used as a tool to reproducibly identify elastic modulus heterogeneities due to the collagen component at the microscale in tissue sections from different organs and species. Using this technique, collagen-enriched domains are distinguished from areas of low collagen content, based on the differences in elastic modulus, in thin (10 μm) sections from human and mouse tissues. The micromechanical measurements correlate well with the amount and location of collagen, information extracted from the optical image of an adjacent section, stained for collagen and used as a reference. Previous studies, based on force volume AFM, have shown weak or no correlations with the collagen component when probes of micrometer size are used^3,4,23^. Our results are obtained through a rapid and robust screening, based on the optimization of both the sample preparation method and the scanning parameters, using a conventional (non high-speed) AFM setup. We demonstrate that a standard AFM, equipped with force volume capability, bright field optical imaging capability and without integrated x–y motorized stage^6,8,22^, can be a practical tool for the characterization of tissues exhibiting a structural heterogeneity, due to uneven collagen distribution.

One of the main objectives of our work is to define a sample preparation method where micromechanical differences due to the tissue composition are preserved, guaranteeing stability of the tissue samples in time and biosafety. For AFM studies, fresh^4,5^ and frozen non-fixed^3,14^ tissue samples are used routinely. Fresh samples, especially those coming from soft organs (liver, kidney) generally exhibit poor stability and create difficulties related to transportation, preservation of the tissue integrity, time constraints during experiments. In this work we use thin histological tissue sections which constitute an ideal geometry for AFM studies and allow for direct correlation analysis with an adjacent stained section. We generated data from Sects. 10 μm thick, collected from tissues prepared following the methods described in the paper and performed comparison of the results. The sample preparation method we propose, based on fresh cryo-sections fixed for a short period of time (10 min), provides conditions preserving not only the tissue micromechanical heterogeneities (higher elastic modulus of the collagen component compared to the remaining extracellular matrix), but also sample stability in time and biosafety. The sample stability allows us to perform multiple experiments and correlation analysis with different biomarkers without the time constraints, imposed when using fresh samples. The proposed method can further be tested for the characterization of anomalies in the distribution of collagen and other components of the extracellular matrix in diseases at those scales where nanometer resolution is not critical^25,26^. We used 10 μm cryo sections from fresh non-fixed tissues as reference samples, since sections of such thickness cannot be cut from fresh soft tissue.

## Results

### Design of the force volume AFM experiment

In force volume AFM, the probe of an atomic force microscope is brought into physical contact and then separated from the sample surface at a constant rate in each position, during the scanning of a square area of  a defined size. During this vertical movement, the cantilever deflection is recorded together with the probe-sample separation distance and converted into force vs. indentation curve^27,28^. Elastic modulus, i.e. the value of the Young’s modulus at each position, is obtained by fitting the deformation part of the force curve with contact mechanics models^17,29,30^ or using reference curve data of known elastic modulus^3,31,32^.

**Figure 1:**
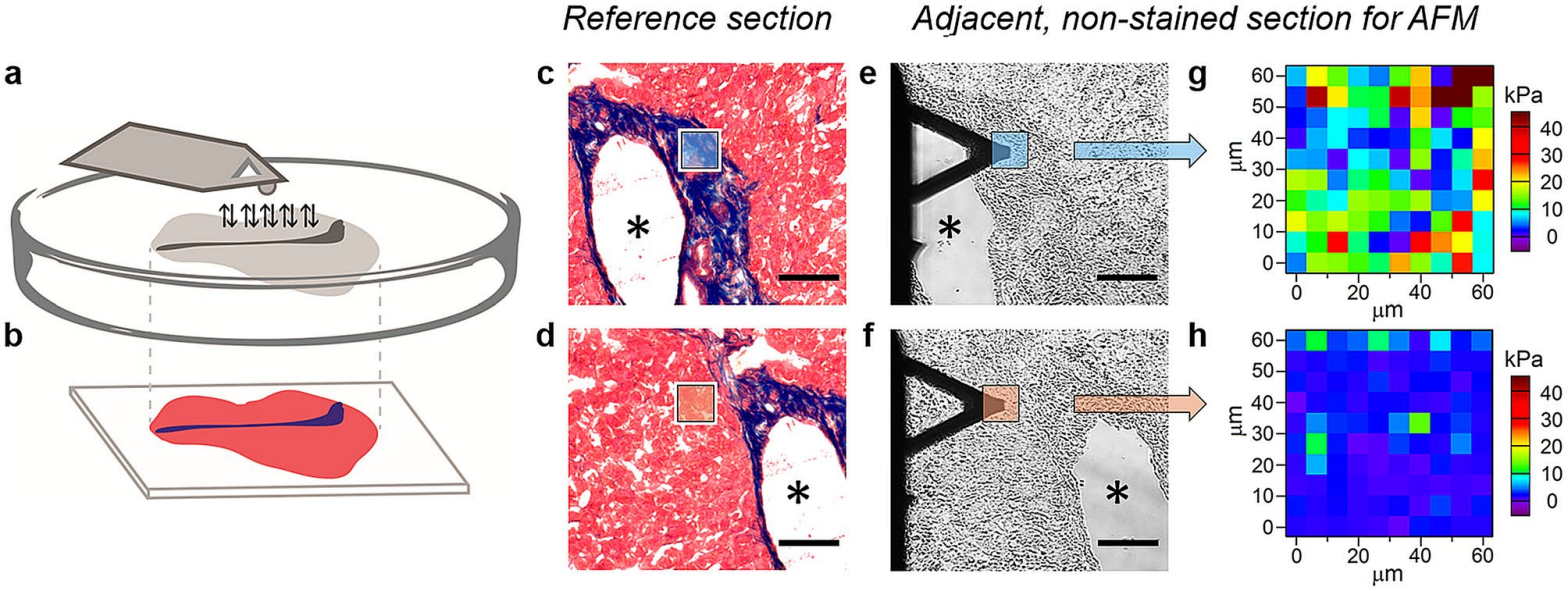
Design of the force volume AFM experiment. (**a**, **b)** Schematic drawing showing the two adjacent sections, the Masson’s trichrome stained and the non-stained section, and the AFM probe. (**c**, **d)**, Histological staining of a human liver tissue section, obtained from a patient with colon cancer (patient #9) (scale bar: 100 μm). In (**c**, **d)**, areas of the tissue with low collagen (CL) (red) and enriched collagen (CE) content (blue) are shown. (**e**, **f)**, Bright field images of the same areas as (**c)** and (**d)** in the adjacent, non-stained section, which has been excised 10 μm far from the stained section (scale bar: 100 μm). The two adjacent sections exhibit similar features, as indicated by the asterisks in the lumen of a large blood vessel in (**c**–**f)**. Images in (**e**, **f)** are obtained during the force volume AFM measurements, using the inverted optical microscope integrated with the AFM system. (**g**, **h)**, Elastic modulus maps of a CE area (**g**) and a CL area (**h**), approximately corresponding to the region highlighted in blue in (**c**, **e)** and in orange in (**d**, **f)**, respectively (maps size: 60 × 60 μm^2^, pixel size: 6 × 6 μm^2^).

Micrometer-sized probes mounted on cantilevers were used in the experiments, conducted on tissue sections from patients (liver) and from mice (liver and kidney) (see the Methods Section). In Fig. 1a, a triangular cantilever with a colloidal probe is schematically depicted, positioned over the surface of a tissue section on a Petri dish. To facilitate the identification of collagen-enriched areas of the sample by force volume AFM, two sections were cut from the tissue block sequentially. One section was stained with Masson’s trichrome stain^33^ and used as a reference for the identification of collagen domains (Fig. 1b), while the adjacent, non-stained section was mounted on the Petri dish for force volume experiments.

It is well known that the sample preparation method is crucial for AFM characterization, especially when mechanical surface properties need to be measured^17^. It would be beneficial to optimize the sample preparation for AFM mechanical characterization through a screening of methods which are mostly recognized and of general use in biology. In our work, we systematically tested four protocols of sample preparation^34^, to obtain: 1) frozen non-fixed, 2) frozen-fixed, 3) fixed-frozen and 4) paraffin sections. Frozen non-fixed sections were collected from a frozen tissue block, embedded in optimal cutting temperature compound (OCT), and measured by force volume AFM without fixation. Frozen-fixed sections were obtained from the same frozen tissue block in OCT, and were fixed in paraformaldehyde (PFA) just before force volume measurements. Fixed-frozen sections were collected from a tissue block, fixed overnight in PFA and then frozen in OCT. Paraffin sections were collected from a tissue block, fixed overnight in PFA and embedded in paraffin. Protocols using frozen non-fixed samples^3,14,31,35–37^ have been largely used for the mechanical characterization with the AFM, while, to our knowledge, there are no reports using paraffin, fixed-frozen and frozen-fixed samples. Deparaffinized and rehydrated tissue sections have been used by Anura et al.^38^ while decellularized ECM has been used by Jorba et al.^39^. All force volume AFM measurements were performed at room temperature and in PBS buffer. More details on the sample preparation methods can be found in the Methods Section.

Human liver samples were obtained from stage IV colon cancer patients undergoing liver resection for colorectal liver metastases and consist of healthy parts of the liver, without metastases (see the Methods Section). Tissue sections prepared with the four different methods were used to determine the elastic modulus in collagen-enriched areas (CE) and areas with low collagen amount (CL). Representative findings from one of the samples, a frozen-fixed section from patient #9, are shown in Fig. 1c-h. In the trichrome-stained section, CE areas, 10–100 µm wide, are clearly recognizable by their intense blue color with the Aniline Blue dye of the Masson’s trichrome^33^. The dye identifies connective tissue around large hollow cavities in the section (arterioles, venules, bile ductules)^40^. The blue color is apparent in the images in Fig. 1c-d, taken around a large blood vessel wall, lying approximately in the center of the section. Bright field optical images of the same areas were also collected in the adjacent non-stained section, with the inverted microscope integrated with the AFM system, during force volume measurements (see Fig. 1e-f). CE areas were identified in the non-stained sections by their position around blood vessels and by the different texture, compared to the texture of CL areas (see also Fig. S1 of the Supplementary Information). Elastic modulus maps were acquired in 60 × 60 μm^2^ areas of the non-stained section, in CE and CL locations. Figure 1g-h show the elastic modulus maps of a collagen-enriched region and a region with low collagen amount, respectively. The approximate location of the areas, scanned with the AFM, is highlighted in blue in Fig. 1e for CE and in orange in Fig. 1f for CL, and the corresponding positions in the stained section are highlighted in Fig. 1c-d, respectively.

By comparing the maps in Fig. 1g and h, we observe that the average elastic modulus of the CE area is higher than the average elastic modulus of the CL area (around 5 kPa). Data from CE areas also show bigger dispersion compared to CL areas. It is worth pointing out that in our work we used colloidal probes of controlled size (tip diameter: 5 µm), which are ideal to map large sample domains in the micrometer range and justify the use of the Hertz model for elastic modulus extraction^17,32,41,42^.

### Effect of the sample preparation method on the mechanical properties of tissue sections

**Figure 2:**
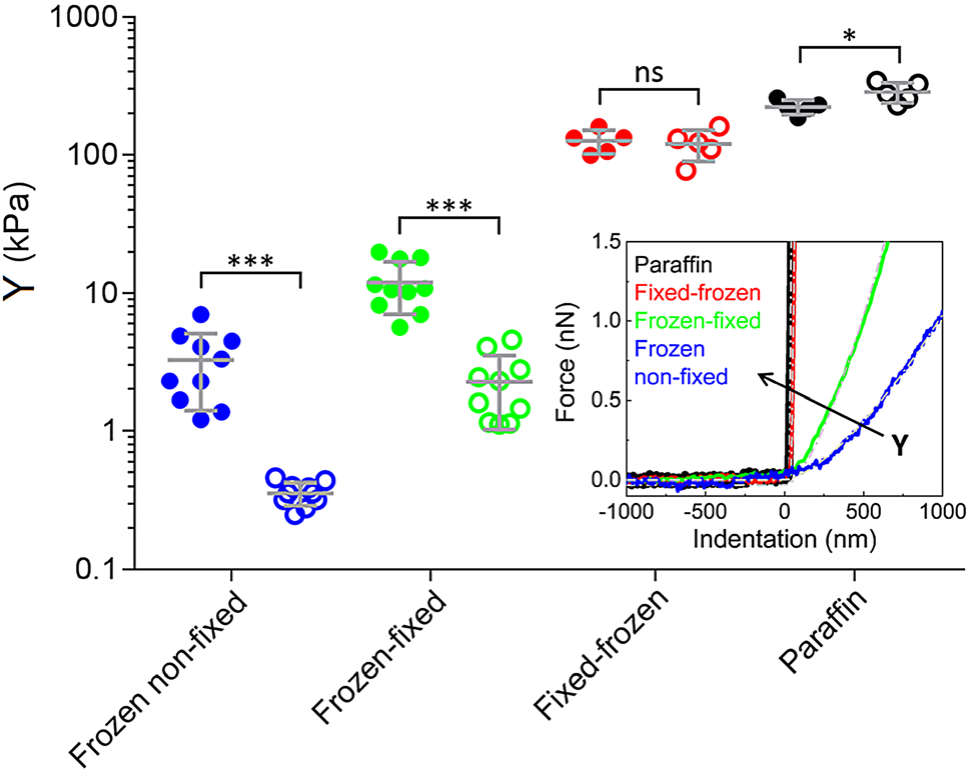
Effect of the preparation method on the elastic modulus of tissue sections. Elastic modulus of CE areas (solid circles) and CL areas (open circles) for each patient in different preparation methods (N = 10 for frozen non-fixed sections and for frozen-fixed sections, represented as blue and green circles, respectively. N = 5 for fixed-frozen sections and for paraffin sections, represented as red and black circles, respectively). Each data point in the graph is an average value for patient, obtained from sampling 5 CE and 5 CL locations with 10 × 10 pixel maps each. Overlapped is the pooled mean with uniform samples and standard deviation. CE locations showed higher elastic modulus in frozen non-fixed and frozen-fixed samples (*P* < 0.001), CE and CL locations showed not significantly different Young’s modulus in fixed-frozen samples (*P* > 0.05) and CL locations showed higher Young’s modulus than CE locations in paraffin samples (*P* < 0.05). Inset. Representative force vs. indentation curves in CL locations for each preparation method. Curves belong to patient #6. The elastic modulus of differently processed tissues, extracted according to the Hertz model, follows the trend: Y_frozen non-fixed sections_ < Y_frozen-fixed sections_ < Y_fixed-frozen sections_ < Y_paraffin sections_. The arrow shows the direction of increasing Young’s modulus.

**Figure 3:**
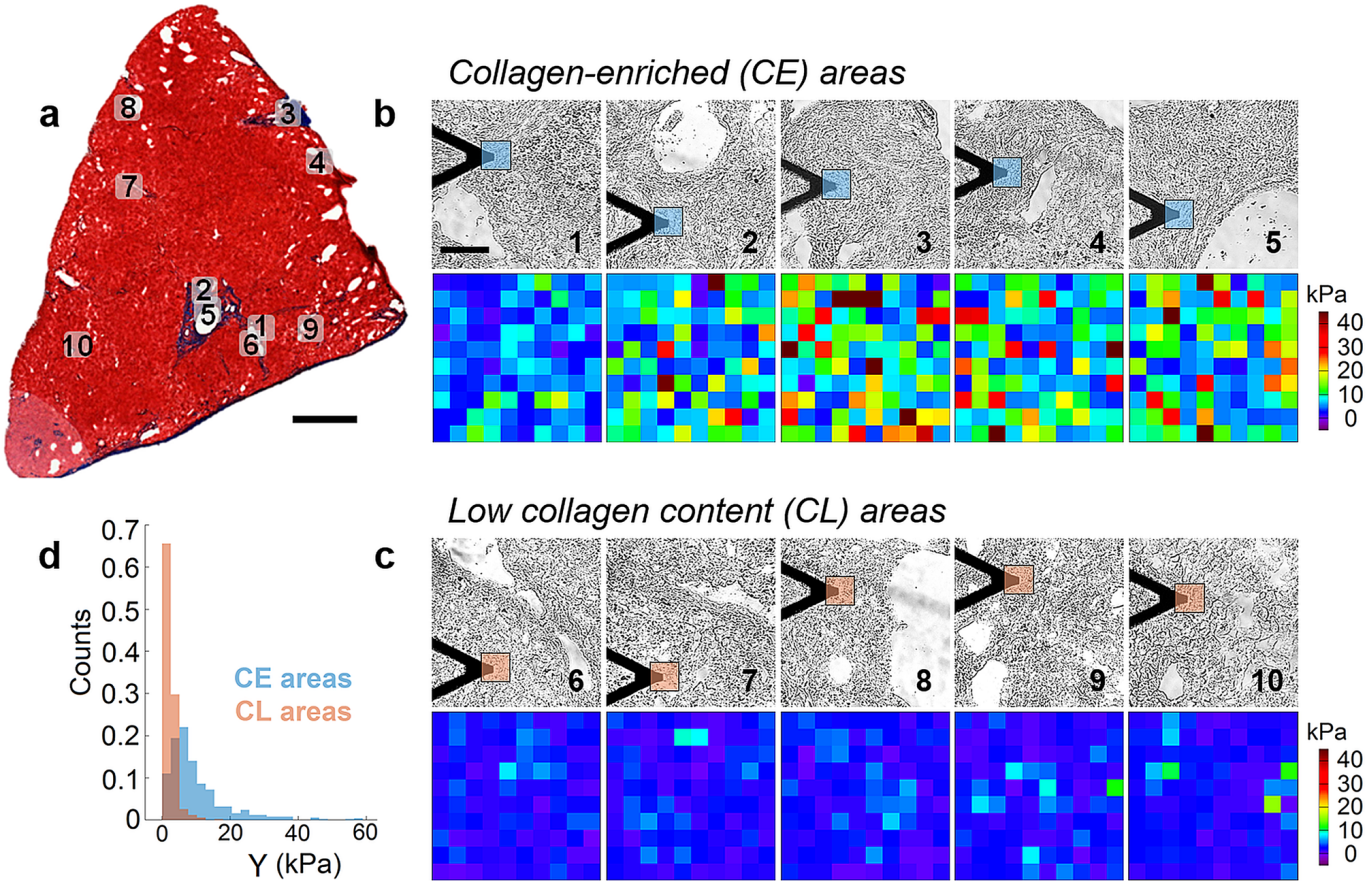
Force volume AFM of frozen-fixed sections from human liver. (a) Reference Masson’s trichrome-stained section of patient #3 (scale bar: 1 mm). (**b**, **c)** (top panels), bright field optical images of the non-stained section, collected during the force volume experiment, corresponding to regions of interest for the CE component (**b**, top panel) and for the CL component (**c**, top panel) of the section, respectively. All 10 regions of interest are highlighted in (**a)**. Scale bar for all the images is 100 μm. (**b**, **c)** (bottom panels) Set of elastic modulus maps (60 × 60 μm^2^, 10 × 10 pixels) from CE (**b**, bottom panel) and CL areas (**c**, bottom panel). The approximate position of the maps is highlighted in blue and orange in (**b** and **c)** (top panels), respectively. (**d)** Distribution histogram of the elastic modulus values plotted separately for CE (N = 500) and CL (N = 500) areas.

**Figure 4:**
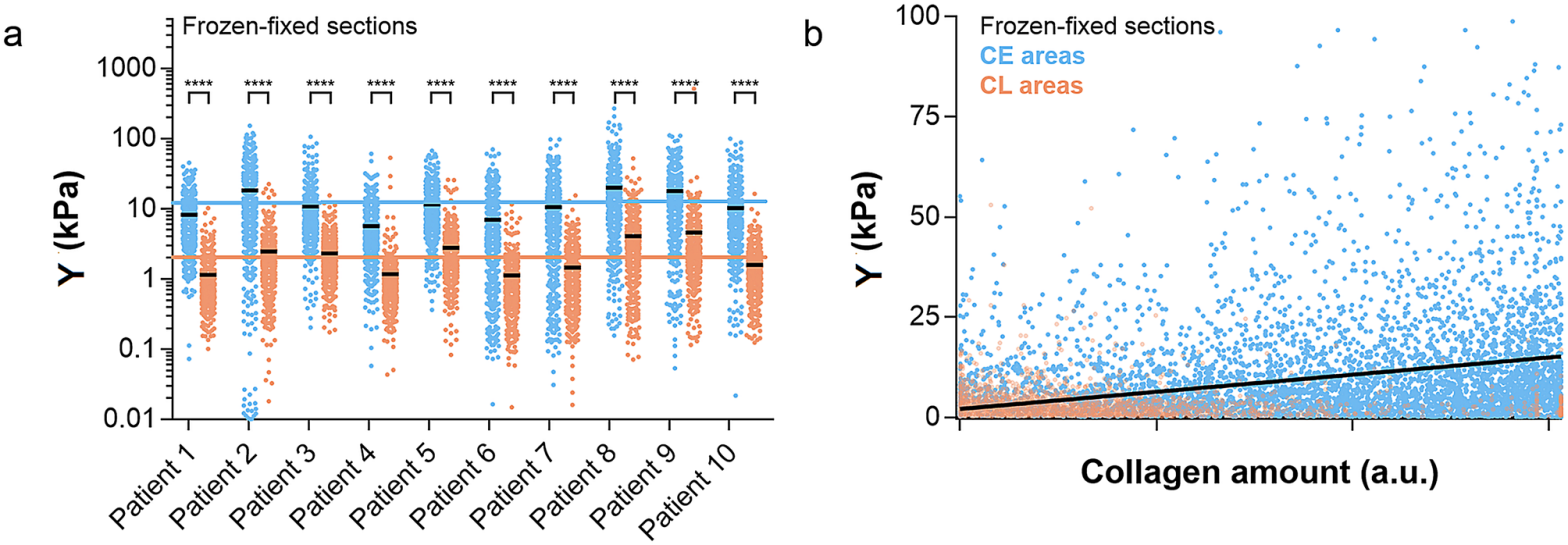
Correlation between elastic modulus and collagen amount**.** (**a**) Elastic modulus values for each patient, plotted separately for CE and CL, with average values (in black). For better visualization, data are shown in logarithmic scale (8 data points are outside the y axis limits). Pooled mean with uniform samples among all patients is also shown in the graph (10.9 kPa for CE, blue line and 2.0 kPa for CL, orange line). Difference between CE and CL elastic modulus was found to be significant for each patient (*P* < 0.0001) and for the all the patients combined (*P* < 0.001, see Fig. 2). (**b**) Correlation between elastic modulus and collagen amount, i.e. the blue intensity in the stained sections, (r_Pearson_ = 0.33, *P* < 0.0001). The linear fit, shown as a black line in (**b)**, includes all CE and CL data as a single population.

In Fig. 2, the results of the AFM micromechanical characterization using the four different preparation methods are shown. For frozen non-fixed and frozen-fixed sections a relatively large sample size was chosen, i.e. ten patients (patients #1–10) were tested. For fixed-frozen and paraffin sections, five patients (patients #6–10) were tested. Composite images containing the trichrome staining, bright field images and elastic modulus maps are shown for each preparation method in Fig. 3 and in Fig. S2-S4. In all frozen non-fixed and frozen-fixed sections, CE areas appeared with significantly higher Young’s modulus than CL areas (*P* < 0.001). Compared to CL elastic modulus, CE elastic modulus was 11.5 times higher in frozen non-fixed sections and 5.5 times higher in frozen-fixed sections (ratio between the pooled mean among all patients is compared, see Fig. S5 and Fig. 4a).

Among all preparation methods, frozen-non-fixed sections are the most similar to native samples. These sections exhibited on average the lowest elastic modulus and the highest difference between CE and CL regions (3.89 kPa and 0.34 kPa respectively, see also the histograms in Fig. S6-S7). Analyzed individually, CE areas showed significantly higher Young’s modulus than CL areas also for each tested patient (*P* < 0.0001) (see Fig. S5). The nominal collagen elastic modulus extracted from frozen non-fixed sections is about two orders of magnitude lower than the elastic modulus of collagen fibrils (a value of 5–10 GPa has been reported for single fibrils from rat tails, measured by nanoindentation in air)^43^ and about one order of magnitude lower than the elastic modulus of articular cartilage, whose main components are collagen and proteoglycans (1–3 MPa, as measured in fresh and frozen tissues from porcine in PBS, using micrometer-sized probes)^5,31^.

In paraffin and fixed-frozen samples, the higher elastic modulus expected for collagen-enriched areas^44^ is no longer detactable by force volume AFM. As expected, due to the long fixation time in PFA, both paraffin and fixed-frozen sections showed the highest elastic modulus among all preparation methods. The inability of identifying collagen by a higher elastic modulus after testing paraffin and fixed- frozen sections from 5 patients by the AFM justifies the choice of a smaller sample size for these types of sections (see also Fig. S8). The average elastic modulus of each sample and method, without dividing to CE and CL areas, is shown in Fig. S9, to help understand the different outcomes of the proposed methodologies. The plot in Fig. S9 can be used as guidance, since the measurements were performed with different probes (paraffin and fixed-frozen samples required stiffer probes with higher k, see the Methods section).

The difference in elastic modulus caused by the different preparation methods is confirmed in single force vs. indentation curves (Fig. 2, inset), collected in single points within CL areas (data are from patient #6). Data fitting with the Hertz model^15,28,32,41,42^ shows that the Young’s modulus is lowest in the frozen non-fixed section (blue curve, Y = 0.39 kPa), followed by the frozen-fixed section (green curve, Y = 1.18 kPa), then the fixed-frozen section (red curve, Y = 115.8 kPa) and highest in the paraffin section (black curve, Y = 309.5 kPa).

The results from the force volume AFM characterization shown in this work are consistent across sections, spaced hundreds of micrometers apart, within the same tissue block. Frozen non-fixed sections, separated by about 200 μm, were investigated, see also the Methods Section. Elastic modulus range and values in CE and CL areas were similar (see the results from patient #7 in Fig.S10a-S10b of the SI). Frozen-fixed sections from mouse liver from levels 200 μm far apart were also compared, showing similar results (Fig. S10c-S10d). These results confirm the robustness of both sample preparation methods. Thus, each of the two methods can be employed as a standard for obtaining reproducible mechanical data with the AFM. We point out that, differently from other approaches^4,45–47^, in our experiments the tissue sections were not glued on the petri dish, in order to avoid the uncontrolled glue deposition that would affect the elastic modulus of the sections.

### Mechanical properties of frozen-fixed tissue sections and correlation with the collagen amount

Our main focus is on the results, obtained from frozen-fixed sections. Figure 3 shows force volume AFM measurements of a liver section, obtained from patient #3. Five regions in each section, with marked CE domains (areas from 1 to 5 in Fig. 3b, top panel) and five regions in the same section, far from the CE locations (CL areas from 6 to 10 in Fig. 3c, top panel) were chosen and optical images of these regions were captured *in-situ* during the force volume experiment. The areas selected for AFM scanning are indicated in the trichrome-stained reference section, as shown in Fig. 3a. The position of the cantilever marks the approximate location of the force maps, highlighted in blue for CE and in orange for CL. In all collected maps (bottom panels in Fig. 3b and 3c) we systematically observed higher elastic modulus in collagen-enriched areas.

Figure 3d shows the distribution histogram of the elastic modulus values, separately from CE and CL locations. It includes data from all the maps, obtained from patient #3. The elastic modulus distribution for CE areas shows a peak at about 6 kPa, while the peak position for the CL areas lies at about 2 kPa. Furthermore, the CE histogram is skewed to high values (60 kPa > Y > 15 kPa), unlike the histogram from the CL areas. Elastic modulus distributions showed similar features for all the tested patients (see Fig. S6 and S7).

Our data for the elastic modulus of all frozen-fixed human sections are presented in Fig. 4a. In this preparation method, the difference between CE and CL data was significant for each individual patient also (*P* < 0.0001). Thus, in frozen-fixed sections from human liver, the collagen component of the tissue can be clearly recognized by force volume AFM due to the higher elastic modulus.

Pixel by pixel correlation analysis between force volume AFM data and histological data (60 × 60 µm^2^ regions of interest in the trichrome sections, ROIs) was also performed for the frozen-fixed sections (see the Methods Section). The results are shown in Fig. 4b, which includes data from all the patients. We found a significant positive correlation^48,49^ between elastic modulus and collagen amount, i.e. the intensity of the blue staining^50,51^, considering CE and CL data as a single dataset (r_Pearson_ = 0.33, *P* < 0.001). Regions with CL content were also included in the analysis, as a contribution from the blue component is present in the trichrome section from these regions. CL (orange) points in Fig. 4b lie in the region of the graph where the collagen amount and elastic modulus are the lowest.

From the results shown in Fig. 4a and 4b, it becomes clear that not only is the elastic modulus in CE areas significantly higher than the elastic modulus of CL areas (see also Fig. 3), but the elastic modulus tends to increase with the amount of collagen (blue intensity) in the reference histological section. The correlation with the collagen amount, demonstrated here for the first time, is relevant, given the use of low-resolution elastic modulus maps (10 × 10 pixels), the physically different sections used for correlation analysis, the positional errors of the measurements and the non-linear relationship between the collagen amount and staining intensity^50,51^. In previously published data, no correlation has been found between AFM micro-elastic modulus and the collagen amount from biochemical assays^3^.

### General applicability of the preparation method based on frozen-fixed sections and spatial correlations

**Figure 5:**
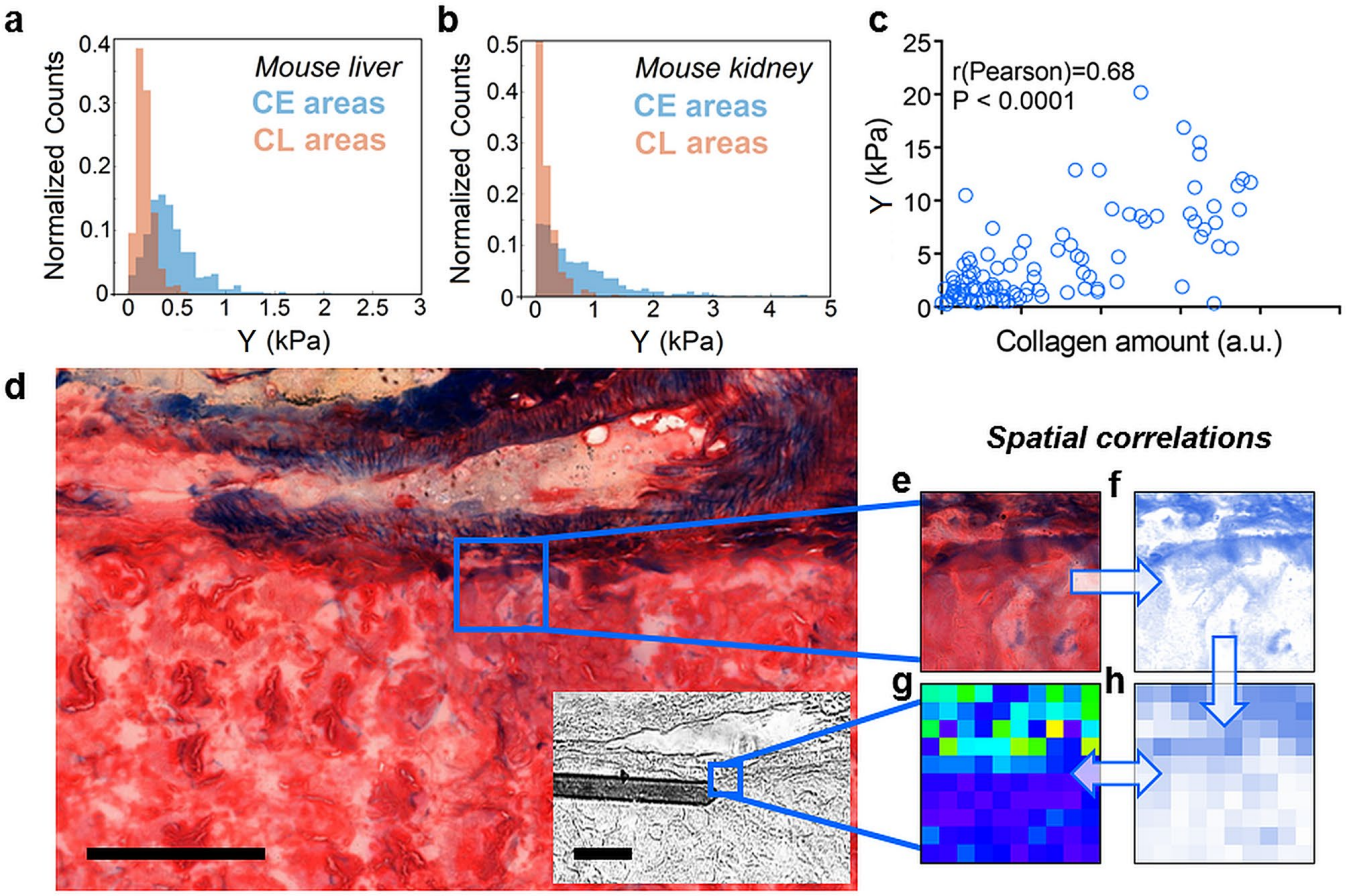
Elastic modulus of frozen-fixed mouse tissue sections and spatial correlations. (**a**, **b)** Young’s modulus distribution histograms of frozen-fixed tissue sections from mouse liver (**a**) and mouse kidney (**b**) plotted separately for CE and CL data (N = 500 for CE and N = 500 for CL). (**c**) Elastic modulus vs. collagen amount (blue intensity), as obtained by comparing pixel by pixel the tiled map from the histological ROI which gave the highest score in the spatial correlation analysis (r_Pearson_ = 0.68, *P* < 0.0001, N = 100) and the low-resolution elastic modulus map (see images in **g**, **h**). (**d)** Trichrome-stained image (**d**) and bright field image (**d**, inset) of the same sample region around a blood vessel in two adjacent sections from mouse kidney (scale bars: 100 mμ). The area where the force map was collected (50 × 50 μm^2^, 10 × 10 pixels) is highlighted in blue in (**d**, inset). (**e–h**) Procedure for spatial correlation analysis (see the main text).

To assess the general applicability of the preparation method based on frozen-fixed sections for collagen identification and evaluate spatial correlations, we performed force volume AFM measurements of frozen-fixed sections from mouse tissues. Figure 5a and 5b show the elastic modulus distribution histograms of a 10 µm thick section from mouse liver (Fig. 5a) and mouse kidney (Fig. 5b) (for both samples, histological staining, bright field optical images and force maps are shown in Fig. S11-S12 of the SI). While the absolute elasticity is tissue-dependent, i.e. sections from mouse kidney appeared to have about two times higher Young’s modulus compared to sections from mouse liver^52^, the elastic modulus of CE areas is higher, compared to the elastic modulus of CL areas in both cases. In mouse liver, the distribution histogram of the CE areas shows a peak at higher elastic modulus (Y ~ 0.4 kPa), compared to the peak of the histogram from CL areas (Y ~ 0.2 kPa), and it shows larger dispersion (Fig. 5a). In mouse kidney, the elastic modulus distribution is skewed to the right (till Y < 5 kPa) in CE areas, while Y values in CL areas lie always below 1 kPa (Fig. 5b). Given the different extent of collagen domains in the two sections, the histogram in Fig. 5a has been obtained with 30 × 30 μm^2^ force volume maps, while the histogram in Fig. 5b has been obtained with 60 × 60 μm^2^ force volume maps.

Figure 5c-h show the spatial correlation between elastic modulus and histological data for a tissue section from mouse kidney. A region of 50 × 50 μm^2^ at the CE-CL interface close to a blood vessel was chosen for the force volume characterization. The position of the cantilever was recorded at the beginning and at the end of the experiment (Fig. S13a-S13b). The optical image of the blood vessel in the reference stained section is shown in Fig. 5d, while the area scanned by the AFM is highlighted in blue in the inset of Fig. 5d. Correlation analysis was performed between the collected elastic modulus map (Fig. 5g) and corresponding, but not identical 50 × 50 µm^2^ ROIs, lying at the CE-CL interface in the stained reference section. The procedure is shown in Fig. 5e–h. Each ROI was color deconvoluted (Fig. 5f) and a new tiled ROI, 10 × 10 pixels was generated, representing the average intensity of the blue component in each pixel (Fig. 5h). The tiled ROI was then correlated pixel by pixel with the map of the elastic modulus, as in Fig. 5c. The highest correlation was obtained with the area highlighted in blue in Fig. 5d (r_Pearson_ = 0.68, *P* < 0.0001). We verified that obtained Pearson’s coefficient was not affected by slight rotations of the optical image of the stained section by ± 5 degrees. For details of the spatial correlation analysis, see also the Methods Section and Fig. S14.

Our results show a significant positive correlation^48,49^ between the tiled ROI from the histological region marked in Fig. 5d and the low-resolution elastic modulus map in Fig. 5g. Previously, only weak spatial correlations (r_Pearson_ = 0.13, *P* < 0.05) were found between elastic modulus maps with high number of pixels (64 × 64 pixels) and corresponding multiphoton microscopy images of the same areas in tendon tissue (same section, no adjacent sections), containing CE and CL domains^3^. We attribute this discrepancy to the different tissues used, since tendon is an intrinsically and overall afibrous tissue and may display higher homogeneity in the mechanical properties under the AFM probe.

To further investigate, a 24 × 24 pixels elastic modulus map was collected in the same area as in Fig. 5d (inset) and correlated with the histological ROI marked in Fig. 5d (see Fig. S15 of the SI). A slightly lower correlation coefficient was obtained, i.e. r_Pearson_ = 0.56, *P* < 0.0001. It is possible that in maps with high number of pixels, features typical of the non-stained section and distinct from the reference section become more prominent and contribute to the decrease in spatial correlation. The time required to collect the high-resolution map in Fig. S15 is 10 min, while only 1.8 min are required to collect the low-resolution map in Fig. 5g.

## Discussion

Despite prevalent use of force volume AFM for the micro and nanomechanical characterization of cells^53,54^, microorganisms^15,55,56^ and viruses^57–59^, relatively little work has been done to measure the mechanical properties of tissues and relate these measurements to function. The lack of experimental data has been justified by the limited availability of samples and sample preparation techniques for subsequent AFM testing^17^. Complementary optical techniques are also necessary with large tissue samples, to guide the AFM probe at the locations of interest and correlate elastic modulus measurements with biochemical reactions, position of fluorescent markers, healthy or disease-affected areas of the same tissue Sect. ^16^ In principle, the samples studied by the AFM should retain tissue morphology, structure and function close to the natural state^60,61^. So far, force volume AFM measurements have been performed with fresh^4,5,45–47^ and frozen non-fixed^3,14,31,35–37^ tissues sections and blocks. The general disadvantage of these samples is the poor stability of the tissue over time and the risk of probe contamination during measurements. There could also be serious safety/health concerns for the researchers, performing the force volume AFM experiments, the equipments and the entire laboratory (pathogens survival, contamination), especially when fresh samples with disease-related mechanical phenotypes are being analyzed. These issues can be overcome by a short-time fixation of cryosections for 10 min before force volume experiments are performed. As expected, the average elastic modulus of the frozen-fixed sections is higher compared to that of frozen non-fixed sections^61^. However, our results inequivocally indicate that the micromechanical differences in elastic modulus, due to the collagen component, are well preserved in the frozen-fixed sections. Furthermore, the frozen-fixed sections remain stable over time and generate similar results after being stored at 4 °C for several days (see Fig. S16).

Here, we present a design of the force volume AFM experiment, aimed to perform a fast tissue characterization with a standard, non- high-speed AFM setup^6,8,18–22^. In our experiments, about 30 min are required for characterizing one tissue section, i.e.for the tip positioning in relevant areas of the sample and the mapping of 10 areas of the section, 5 for CE and 5 for CL locations. This short timescale is achieved by efficient probe positioning, performed under the guidance of the stained reference section, and by optimization of the scanning parameters (speed of a single approach-retraction curve, number of pixels/map). The collection of bright field images in situ during the force volume AFM experiment facilitates the identification of the elastic modulus maps within the optical field of the measurements, and speeds up the subsequent data processing and correlation analysis. From our results, differences in elastic modulus due to the collagen component are observed in low resolution maps, whose size is commensurate with the size of the collagen domains in the sections from different organs (60 × 60 µm^2^ in human liver and mouse kidney, 30 × 30 µm^2^ in mouse liver). The data shown here have been obtained by setting the speed of a single approach-retraction cycle (force vs. distance curve) to 1 Hz. We found that increasing the rate of the force curves collection to 1.5 Hz does not reduce the sensitivity of the measurements nor affects the quality of the results obtained. The differences in elasticity between CE and CL areas, absolute Young’s modulus values for each preparation method, force curve evolution and fitting results remain unchanged with the increase of the scanning rate from 1 to 1.5 Hz (data not shown). The higher imaging speed further reduces the overall duration of the force volume AFM experiment by about 30% for a single section. The force volume AFM can become an integral part of biological experiments and a useful tool in the clinical practice to obtain micromechanical fingerprints of tissues, label-free, with high reproducibility and affordable timescales. Previous reports of tissues elastic modulus by force volume AFM showed slower outcomes, 1 to 2 orders of magnitude, and the time necessary for the collection of a single map varied between 10 and 100 min^3,4^.

We observed differences in the elastic modulus between different patients in frozen non-fixed and in frozen-fixed sections. In general, differences (both CE and CL values) were significant (Table S1). Since all tissue samples were obtained from normal, non-metastatic areas of the patients ‘ liver, we attribute these differences to different tissue phenotypes. Future studies will focus on the evaluation of subtle differences between patients, correlation with progression of the disease, anomalous collagen distribution or the presence of metastases^12^.

In conclusion, in our work we implement the force volume AFM to study elastic modulus gradients in tissue sections at the microscale. Using collagen as an example, we screened different sample preparation methods for their potential to measure precisely the effect of collagen content on the mechanical properties of the section. Together with the standardization of the sample preparation method, we present a standardization of the AFM measurements for fast scanning, allowing generation of reproducible results. The experimental design includes preparation of a reference stained section for correlation analysis. Our preparation method, based on frozen-fixed sections, can be further tested in dedicated setups for high-speed IT-AFM using motorized stages^6,8,22^ or faster designs for the z-scanner movement^62^, in order, for example, to assess the overall quality of the sections, detect micromechanical heterogeneities due to tissue components other than collagen, understand the origin of elasticity at the single molecule level or unveil more subtle differences between patients, effects of treatments, etc., data that only the AFM can capture, due to its extremely high sensitivity and spatial resolution. An important additional benefit of our method is that tissue with preserved quality and stability allows for AFM studies to be correlated with different in situ molecular detection methodologies, and for mechanical measurements to be integrated with other studies of interest for extended periods of time.

## Methods

All methods were carried out in accordance with relevant guidelines and regulations. For human samples, all individuals provided informed consent for liver tissue biopsy. The experiments with the human tissues were approved by the Memorial Sloan Kettering Institutional Review Board (MSK- IRB protocol #17–594). The experiments with the mouse tissues were approved by the Memorial Sloan Kettering Institutional Animal Care and Use Committee (MSK-IACUC protocol #01–11-026).

### Preparation of the tissue sections

Non-tumoral human liver samples were obtained from patients with stage IV colon cancer undergoing resection of liver metastases at Memorial Sloan Kettering Cancer Center (MSK). Prior to getting the liver biopsies in the operating room (OR), doctors reviewed the computed tomography scans to identify where the metastatic liver lesions are and selected an area in the liver that appears normal. The surgeon then biopsied that area that appeared grossly normal non-metastatic (as far as possible from the liver lesion). A sample of this biopsy was then fixed and stained for histological analysis to confirm that it was normal liver tissue. Upon specimen collection, liver tissue was placed into ice-cold PBS and transported immediately from the OR to the laboratory for processing. Protease inhibitors were not added to PBS^3,14,35,36^, as only part of the tissue was used for the AFM measurements. The remaining tissue was used for other tests (exosome isolation, tissue culture), which require preserved cell viability. Due to the limited duration of the AFM experiments, we do not expect that the cell enzymatic activity can affect the AFM measurements. Liver tissue from each patient was cut into three parts. Each part was processed in one of the following ways: paraffin embedding, fixed-frozen, and frozen preparations. For paraffin and fixed-frozen preparations, tissues were immersed in 4% paraformaldehyde (PFA) and fixed overnight at 4 °C with rocking, then washed three times with PBS. The processing of the tissues for embedding in paraffin continued with a tissue processor (Leica Biosystems, ASP6025) as follows: dehydration in 95% ethanol for 30 min and in 100% ethanol twice for 30 min each, clearing in xylene 3 times, 30 min each. The tissues were then immersed in liquid paraffin (3 times for 30 min each, T = 56 °C). The paraffin blocks were stored in the refrigerator. 10 μm thick paraffin sections were cut with a microtome (Leica RK2065), mounted on the glass bottom Petri dishes, dewaxed and rehydrated. For fixed-frozen preparation, after fixation and washing in PBS, the tissues were transferred in 30% sucrose^63,64^ and rocked at 4 °C until the tissues rested on the bottom of the vial. The tissues were transferred in equal volumes of OCT and 30% sucrose, rocked for 2–3 h at 4 °C, equilibrated in OCT for 30 min at 4 °C and embedded in molds filled with OCT. The molds were quickly frozen in isopentane, pre-cooled in liquid nitrogen and stored in a freezer at T = -80 °C. For frozen non-fixed and frozen-fixed preparation, the tissues were embedded in OCT, frozen and stored as described for the fixed-frozen samples. For all cryo-preparations, four 10 μm thick sections (Sect. 1–4) were cut sequentially with a cryostat (Leica Biosystems, CM1950). Sections 2 and 3 were transferred in a glass bottom Petri dish (FluoroDish FD5040), coated with poly-L-lysine (1 × dilution from 10 × stock solution, P8920 Sigma for 30 min at RT) and kept on ice until the start of the force volume AFM experiment (only one section was scanned). Section 1 (reference for Sect. 2) and Sect. 4 (reference for Sect. 3) were stained with Masson’s trichrome stain (see below). The frozen non-fixed sections were scanned as soon as possible. For the frozen-fixed preparation, cryo-sections from frozen non-fixed blocks were fixed for 10 min with 4% PFA on ice, washed with cold PBS and scanned. All reagents, solvents and Petri dishes used in this work were obtained from Fisher Scientific. Frozen-fixed sections from wild type C57Bl/6 mouse liver and kidney tissues were prepared in the same way as the human frozen-fixed sections.

### Masson’s trichrome staining

Sections 1 and 4 from each of the four different types of tissue preparations were mounted on a Superfrost Plus glass slides (Fisher Scientific, 12–550-15). The slides were baked at 58 °C for 60 min and stained with Masson’s trichrome stain (Abcam, ab150686) according to the manufacturer instructions. The slides were digitally scanned with the Panoramic Flash 250 scanner (3DHistech, Budapest, Hungary), using 20x/0.8NA Zeiss objective or Zeiss Lumar v.12 stereomicroscope. The regions of interest (ROIs) were manually drawn on the scanned sections, using CaseViewer software (3DHistech, Budapest, Hungary), exported into .tiff files at full resolution (0.243 mm/pixel) and analyzed using ImageJ (NIH) or MATLAB (MathWorks).

### Force volume AFM experiment and force curves analysis

Glass bottom Petri dishes containing freshly cut tissue sections were filled with 3 ml PBS and scanned with the AFM. Experiments were performed with a MFP-3D-BIO AFM (Oxford Instruments), integrated with an inverted Zeiss AxioObserver Z1 microscope and an AxioCam. The Zeiss microscope was used to visually position the AFM cantilever with respect to the sample. The positions for AFM scanning on the unstained section were chosen based on the image of the trichrome stained referenced section. Cantilevers with colloidal probes were used in this work. For the frozen-fixed and frozen non-fixed sections, borosilicate (BS) probes with 5 μm diameter and nominal k = 0.09 N/m were used (Novascan), while for the paraffin and fixed-frozen sections, polystyrene (PS) probes with 6.1 μm diameter and nominal k = 0.02–0.77 N/m were used (CP-CONT-PS, NanoandMore). Before each experiment, the cantilever spring constant was calibrated using the thermal noise method^65^ and the optical sensitivity was determined using a glass bottom Petri dish, filled with PBS, as an infinitely stiff substrate. AFM was performed at RT by mapping regions within the area of the bright field images. The area of the force maps is 60 × 60 μm^2^ (sections from human liver), 30 × 30 μm^2^ (sections from mouse liver), 60 × 60 μm^2^ (sections from mouse kidney) and 50 × 50 μm^2^ (section from mouse kidney used for correlation analysis, see Fig. 5). The rate of a single approach/withdraw cycle was set to 1 Hz. Under these conditions, the mapping of one area took 1.8 min. We verified that decreasing the approach/withdraw rate does not affect the force curve evolution or change the obtained Young’s modulus from curve fitting^17^. A force threshold was chosen to ensure sample penetration of 1–2 microns for all the preparations (force threshold = 10 nN for paraffin sections, 3 nN for fixed-frozen sections, 3 nN for frozen-fixed sections and 3 nN for frozen non-fixed sections). Force curves in each map were fitted according to the Hertz model^15,28,32,41,42^, using the routine implemented in the MFP 3D AFM to obtain elastic modulus maps (Igor Pro, Wavemetrics) (ν_tip_ = 0.5, E_tip_ = 3 GPa, ν_sample_ = 0.45^66^ for PS probes and ν_tip_ = 0.19, E_tip_ = 68 GPa, ν_sample_ = 0.45^66^ for BS probes).

### Dataset and controls

The data presented here were generated from 5 paraffin, 5 fixed-frozen (patients #6–10), 10 frozen non-fixed and 10 frozen-fixed sections (patients #1–10). The data from one frozen-fixed section from mouse liver and one from mouse kidney were also collected. To assess the consistency of the elastic modulus results within the same patient block and the robustness of the preparation method, two additional frozen non-fixed sections from human liver and two additional frozen-fixed sections from mouse liver, 200 μm away from the original section, were also cut and tested (Sects. 2 and 3). To assess the stability of the tissue sections over time, frozen-fixed Sect. 2 from patient #2 was tested immediately after preparation, while the Sect. 3 was stored in PBS at 4 °C and scanned 5 days later (see Fig. S16 of the SI). In Table S2 of the SI the total number of scanned sections is resumed, together with the number of collected force maps/sample, the size of the maps and the resolution (number of pixels/map).

### Data processing

Normalized elastic modulus distribution histograms were obtained from the AFM data through a custom script in MATLAB (MathWorks). Histograms were generated using 100 uniformly distributed bins between a maximum and a minimum set per data set. Normalization was obtained by dividing the data points within a certain bin by the total number of data points and converting the number to a percentage. Data visualization and fitting were performed using OriginPro (OriginLab) and GraphPad Prism. Statistical analyses, i. e. unpaired parametric t-test and Pearson’s correlation analysis were performed with GraphPad Prism. The average elastic modulus is reported as pooled mean across patients.

### Correlation analysis

To correlate collagen staining with elastic modulus in all human samples, regions approximately matching the AFM force maps were extracted from the stained trichrome sections. Manual placement of the fixed size ROIs was guided by the optical images showing the cantilever position and the surrounding tissue, as well as by the elastic modulus maps of sampled locations, made during the force measurements. In most (~ 85%) cases a distinctive tissue feature, such as an edge or void was visible in both optical images and stained adjacent sections allowing relatively unambiguous determination of where the tip was placed during AFM measurements. In the remaining featureless areas of tissue this was not possible, and an approximate region within the adjacent section guided by a coarser scale manual record of sampled locations made at imaging time was used. In many cases features, such as regions of collagen with higher Youngs modulus, were also visible within the force maps, allowing confirmation of the alignment (see Fig. S13 c-d of the SI). Accuracy was limited by the manual procedure, the variability in the cantilever’s position relative to the sampled area at the time brightfield images were acquired and by the physically different stained and non-stained sections.

To measure collagen staining, the trichrome images of the ROIs matching the force maps were color deconvoluted using the default Masson trichrome vector in ImageJ. The resulting blue channel image was inverted. The intensity within all pixels corresponding to a single pixel in the AFM map were averaged to create a single staining value corresponding to each Young’s modulus point (spatial details for human and mouse samples below). The extent of correlation between a given elastic modulus value and corresponding collagen amount was computed using the Pearson’s correlation coefficient.

Only the spatial sampling used to extract collagen values corresponding to each elastic modulus data differs between human and mouse samples. For human samples the total imaged ROI was 60 × 60 μm^2^. All adjacent section pixels within an area of 6 × 6 μm^2^ (one pixel in force maps) were summed and averaged to create the corresponding collagen value. In mouse kidney samples, the total ROI area is 150 × 85 µm^2^. Within this area, 50 × 50 µm^2^ ROIs were extracted with 2-pixel spacing in both x and y directions (1 pixel = 0.12 µm). In this case, all adjacent section pixels within an area of 5 × 5 μm^2^ (one pixel in force maps) were averaged to create the corresponding collagen value.

## Supplementary information

Supplementary file1

## References

[1] Lampi MC, Reinhart-King CA (2018). Targeting extracellular matrix stiffness to attenuate disease: From molecular mechanisms to clinical trials. Sci. Transl. Med..

[2] Insua-Rodríguez J, Oskarsson T (2016). The extracellular matrix in breast cancer. Adv. Drug Deliv. Rev..

[3] Marturano JE, Arena JD, Schiller ZA, Georgakoudi I, Kuo CK (2013). Characterization of mechanical and biochemical properties of developing embryonic tendon. PNAS.

[4] Plodinec M (2012). The nanomechanical signature of breast cancer. Nat. Nanotechnol..

[5] Stolz M (2009). Early detection of aging cartilage and osteoarthritis in mice and patient samples using atomic force microscopy. Nat. Nanotechnol..

[6] Koser DE (2016). Mechanosensing is critical for axon growth in the developing brain. Nat. Neurosci..

[7] Betz T, Koch D, Lu Y-B, Franze K, Käs JA (2011). Growth cones as soft and weak force generators. PNAS.

[8] Barriga EH, Franze K, Charras G, Mayor R (2018). Tissue stiffening coordinates morphogenesis by triggering collective cell migration in vivo. Nature.

[9] Pathak A, Kumar S (2012). Independent regulation of tumor cell migration by matrix stiffness and confinement. PNAS.

[10] Baker BM (2015). Cell-mediated fibre recruitment drives extracellular matrix mechanosensing in engineered fibrillar microenvironments. Nat. Mater..

[11] Riching KM (2014). 3D collagen alignment limits protrusions to enhance breast cancer cell persistence. Biophys. J..

[12] Zemła J (2018). Atomic force microscopy as a tool for assessing the cellular elastic modulus and adhesiveness to identify cancer cells and tissues. Semin. Cell Dev. Biol..

[13] 13Liu, L. *et al.* Mechanoresponsive stem cells to target cancer metastases through biophysical cues. *Sci. Transl. Med.* **9**, eaan2966 (2017). 10.1126/scitranslmed.aan2966 PMC5890431 28747514

[14] Lindeman JHN (2010). Distinct defects in collagen microarchitecture underlie vessel-wall failure in advanced abdominal aneurysms and aneurysms in Marfan syndrome. PNAS.

[15] Touhami A, Nysten B, Dufrêne YF (2003). Nanoscale mapping of the elastic modulus of microbial cells by atomic force microscopy. Langmuir.

[16] Dufrêne YF (2017). Imaging modes of atomic force microscopy for application in molecular and cell biology. Nat. Nanotechnol..

[17] Krieg M (2019). Atomic force microscopy-based mechanobiology. Nat. Rev. Phys..

[18] Kodera N, Yamamoto D, Ishikawa R, Ando T (2010). Video imaging of walking myosin V by high-speed atomic force microscopy. Nature.

[19] Chiaruttini N (2015). Relaxation of loaded ESCRT-III spiral springs drives membrane deformation. Cell.

[20] Ruan Y (2017). Direct visualization of glutamate transporter elevator mechanism by high-speed AFM. PNAS.

[21] Wang A, Vijayraghavan K, Solgaard O, Butte MJ (2015). Fast stiffness mapping of cells using high-bandwidth atomic force microscopy. ACS Nano.

[22] Thompson AJ (2019). Rapid changes in tissue mechanics regulate cell behaviour in the developing embryonic brain. Life.

[23] Özdemir BC (2014). Depletion of carcinoma-associated fibroblasts and fibrosis induces immunosuppression and accelerates pancreas cancer with reduced survival. Cancer Cell.

[24] Levental KR (2009). Matrix crosslinking forces tumor progression by enhancing integrin signaling. Cell.

[25] Zhang K (2013). The collagen receptor discoidin domain receptor 2 stabilizes SNAIL1 to facilitate breast cancer metastasis. Nat. Cell Biol..

[26] Provenzano PP (2008). Collagen density promotes mammary tumor initiation and progression. BMC Med..

[27] Heinz W, Hoh J (1999). Spatially resolved force spectroscopy of biological surfaces using the atomic force microscope. Trends Biotechnol..

[28] Radmacher M, Fritz M, Kacher CM, Cleveland JP, Hansma PK (1996). Measuring the viscoelastic properties of human platelets with the atomic force microscope. Biophys. J..

[29] Roduit C (2009). Stiffness tomography by atomic force microscopy. Biophys. J..

[30] Calò A (2014). Force measurements on natural membrane nanovesicles reveal a composition-independent, high Young's modulus. Nanoscale.

[31] Stolz M (2004). Dynamic elastic modulus of porcine articular cartilage determined at two different levels of tissue organization by indentation-type atomic force microscopy. Biophys. J..

[32] Schillers H (2017). Standardized nanomechanical atomic force microscopy procedure (SNAP) for measuring soft and biological samples. Sci. Rep..

[33] Campbell CB, Cukierman E, Artym VV (2014). 3-D extracellular matrix from sectioned human tissues. Curr. Protoc. Cell Biol..

[34] Suvarna KS, Layton C, Bancroft JD (2019). Bancroft's theory and practice of histological techniques, 98–103.

[35] Sicard D, Fredenburgh LE, Tschumperlin DJ (2017). Measured pulmonary arterial tissue stiffness is highly sensitive to AFM indenter dimensions. J. Mech. Behav. Biomed. Mater..

[36] Van Zwieten RW (2014). Assessing dystrophies and other muscle diseases at the nanometer scale by atomic force microscopy. Nanomedicine.

[37] Zemła J (2018). AFM-based nanomechanical characterization of bronchoscopic samples in asthma patients. J. Mol. Recognit..

[38] Anura A (2017). Nanomechanical signatures of oral submucous fibrosis in sub-epithelial connective tissue. J. Mech. Behav. Biomed. Mater..

[39] Jorba I, Uriarte JJ, Campillo N, Farré R, Navajas D (2017). Probing micromechanical properties of the extracellular matrix of soft tissues by atomic force microscopy. J. Cell. Physiol..

[40] Rogers AB, Dintzis RZ (2018). Comparative anatomy and histology.

[41] Holtzmann K (2016). Brain tissue stiffness is a sensitive marker for acidosis. J. Neurosci. Methods.

[42] Lekka M (2012). Cancer cell detection in tissue sections using AFM. Arch. Biochem. Biophys..

[43] Wenger MP, Bozec L, Horton MA, Mesquida P (2007). Mechanical properties of collagen fibrils. Biophys. J..

[44] Bonnans C, Chou J, Werb Z (2014). Remodelling the extracellular matrix in development and disease. Nat. Rev. Mol. Cell Biol..

[45] Ansardamavandi A, Tafazzoli-Shadpour M, Omidvar R, Jahanzad I (2016). Quantification of effects of cancer on elastic properties of breast tissue by Atomic Force Microscopy. J. Mech. Behav. Biomed. Mater..

[46] Ciasca G (2016). Nano-mechanical signature of brain tumours. Nanoscale.

[47] Tian M (2015). The nanomechanical signature of liver cancer tissues and its molecular origin. Nanoscale.

[48] Akoglu H (2018). User's guide to correlation coefficients. Turk. J. Emerg. Med..

[49] Mukaka MM (2012). Statistics corner: A guide to appropriate use of correlation coefficient in medical research. Malawi Med. J..

[50] Carriel VS (2011). A novel histochemical method for a simultaneous staining of melanin and collagen fibers. J. Histochem. Cytochem..

[51] Oliveira AC (2013). Evaluation of small intestine grafts decellularization methods for corneal tissue engineering. PLoS ONE.

[52] Wells RG (2013). Tissue mechanics and fibrosis. Biochim. Biophys. Acta.

[53] Moeendarbary E (2013). The cytoplasm of living cells behaves as a poroelastic material. Nat. Mater..

[54] Picas L, Rico F, Deforet M, Scheuring S (2013). Structural and mechanical heterogeneity of the erythrocyte membrane reveals hallmarks of membrane stability. ACS Nano.

[55] Alsteens D, Trabelsi H, Soumillion P, Dufrêne YF (2013). Multiparametric atomic force microscopy imaging of single bacteriophages extruding from living bacteria. Nat. Commun..

[56] Gaboriaud F, Bailet S, Dague E, Jorand F (2005). Surface structure and nanomechanical properties of *Shewanella Putrefaciens* bacteria at two pH values (4 and 10) determined by atomic force microscopy. J. Bacteriol..

[57] Marchetti M, Wuite G, Roos W (2016). Atomic force microscopy observation and characterization of single virions and virus-like particles by nano-indentation. Curr. Opin. Virol..

[58] Michel JP (2006). Nanoindentation studies of full and empty viral capsids and the effects of capsid protein mutations on elastic modulus and strength. PNAS.

[59] Hernando-Pérez M (2012). Direct measurement of phage phi29 stiffness provides evidence of internal pressure. Small.

[60] Eisenberg BR, Mobley BA (1975). Size changes in single muscle fibers during fixation and embedding. Tissue Cell.

[61] Hoh JH, Schoenenberger C-A (1994). Surface morphology and mechanical properties of MDCK monolayers by atomic force microscopy. J. Cell Sci..

[62] Chopinet L, Formosa C, Rols MP, Duval RE, Dague E (2013). Imaging living cells surface and quantifying its properties at high resolution using AFM in QI^TM^ mode. Micron.

[63] McDowall A (1983). Electron microscopy of frozen hydrated sections of vitreous ice and vitrified biological samples. J. Microscopy.

[64] Griffiths G, McDowall A, Back R, Dubochet J (1984). On the preparation of cryosections for immunocytochemistry. J. Ultrastruct. Res..

[65] Sader JE, Larson I, Mulvaney P, White LR (1995). Method for the calibration of atomic force microscope cantilevers. Revi. Sci. Instrum..

[66] Chen EJ, Novakofski J, Jenkins WK, O'Brien WD (1996). Young's modulus measurements of soft tissues with application to elastic modulus imaging. IEEE Trans. Ultrason. Ferroelectr. Freq. Control.

