# Supplementary material for: Spatial mapping of the collagen distribution in human and mouse tissues by force volume atomic force microscopy
Source: Sci Rep. 2020 Sep 24;10:15664. doi: 10.1038/s41598-020-72564-9 (PMC7518416; doi:10.1038/s41598-020-72564-9)
Supplement: Supplementary file 1 — Supplementary file1 [file 41598_2020_72564_MOESM1_ESM.pdf]

Supplementary Information

Spatial mapping of the collagen distribution in human and mouse tissues by force volume atomic force microscopy

Annalisa Calò[1,2]*, *Yevgeniy Romin*[1], *Rami Srouji*[3,4], *Constantinos P. Zambirinis*[3,5], *Ning Fan*[1], *Anthony Santella*[1], *Elvin Feng*[1], *Sho Fujisawa*[1], *Mesruh Turkekul*[1], *Sharon Huang*[1], *Amber L. Simpson*[3,6], *Michael D'Angelica*[3], *William R. Jarnagin*[3], *and Katia Manova-Todorova*[1]*

[1] Molecular Cytology Core Facility, Memorial Sloan Kettering Cancer Center, 417E 68th St, 10065 New York (NY)

[2] Physics Faculty, University of Barcelona (UB), Carrer de Martí i Franquès 1-11, 08028 Barcelona (Spain)

[3] Hepatopancreatobiliary Service, Department of Surgery, Memorial Sloan Kettering Cancer Center, 1275 York Ave, 10065 New York (NY)

[4] Washington University School of Medicine, 660 S. Euclid Ave., 63110 St. Louis (MO)

[5] Department of Surgery, Warren Alpert Medical School of Brown University, 593 Eddy street, APC-4, 02903 Providence (RI)

[6] School of Computing, Queen's University, 557 Goodwin Hall, K7L 2N8 Kingston (ON, Canada)

These authors contributed equally: Annalisa Calò, Yevgeniy Romin

*email: annalisa.calo@ub.edu, k-manova@ski.mskcc.org

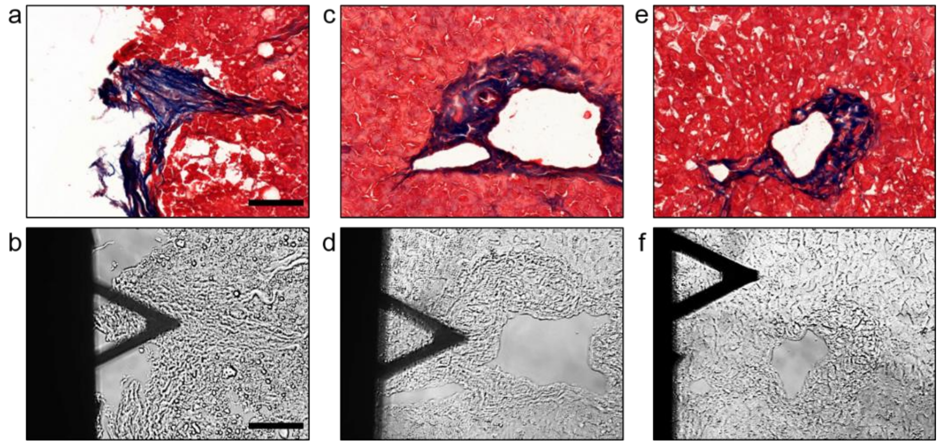

Figure S1. **Images of the same regions in stained and non-stained adjacent sections.** **a**, **c**, **e**, Trichrome staining of liver tissue sections from patient #1 (**a**), patient #7 (**c**) and patient #9 (**e**) (scale bar: 100 μm). **b**, **d**, **f**, Corresponding areas in the non-stained section (scale bar: 100 μm). Collagen-enriched areas, which appear blue in the stained sections, can be recognized in bright field images by their position and the different texture compared to areas with low collagen amount.

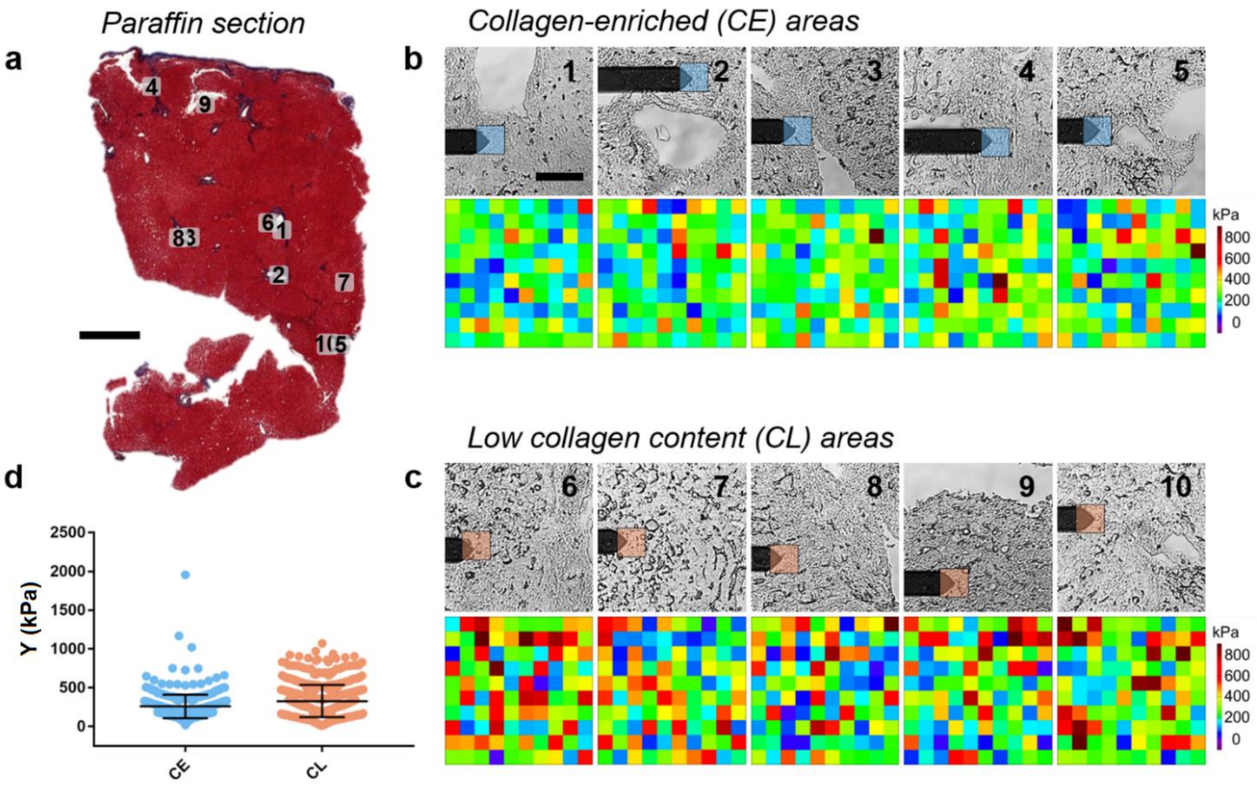

Figure S2. Force volume AFM measurements in paraffin sections from human liver. a, Reference trichrome-stained section from patient #6 (scale bar: 1 mm). **b**, **c** (top panels), Set of bright field optical images of the adjacent, non-stained section collected during the force volume AFM experiment, corresponding to regions of interest for the CE component (**b**, top panel) and for the CL component (**c**, top panel). These areas are highlighted in (**a). ** Scale bar for all images: 100 μm. **b**, **c** (bottom panels), Set of elastic modulus maps (size: 60x60 μm^2, 10x10 pixels) taken approximately in the areas marked in blue in **b** (top panel) and in orange in **c** (top panel). **d**, Elastic modulus values for patient #6, obtained from all the CE and CL maps (5CE/5CL areas, 10x10 pixels/map), with overlapped average values and standard deviation (SD).

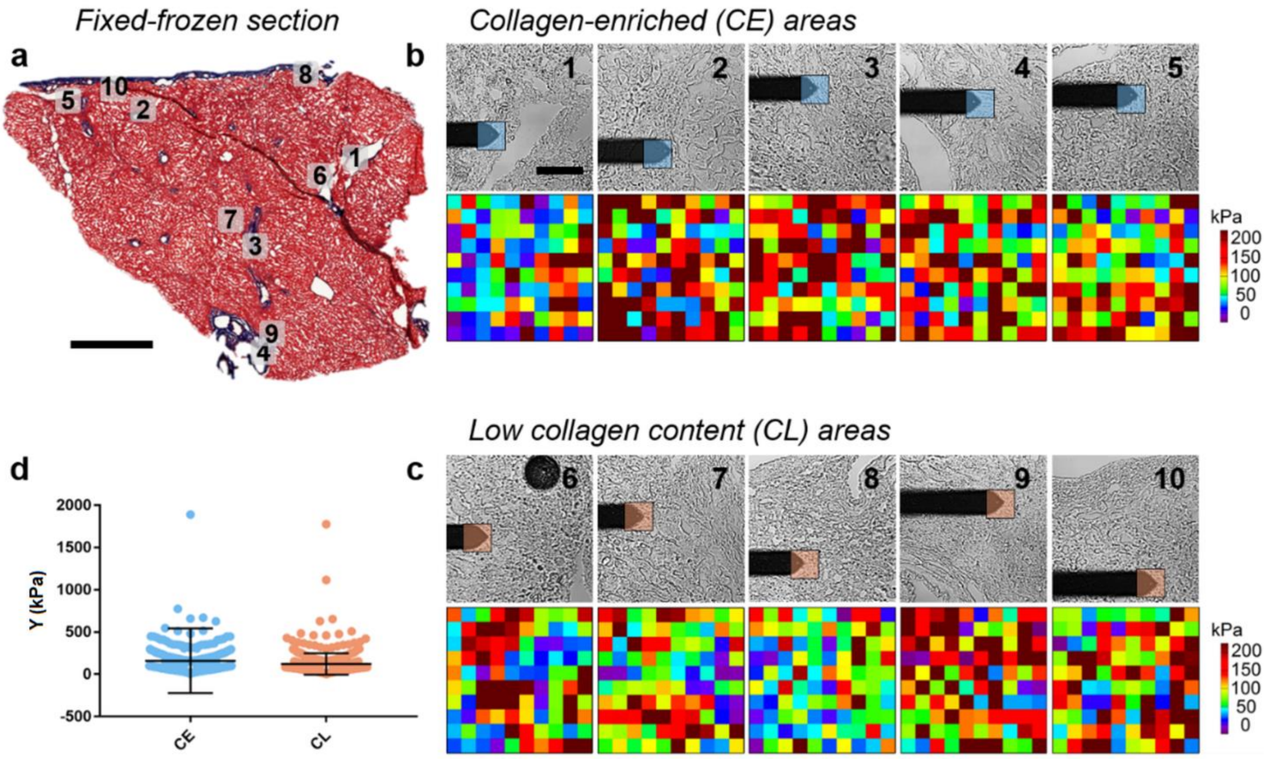

Figure S3. Force volume AFM measurements in fixed-frozen sections from human liver. a, Reference trichrome-stained section (patient #10, scale bar: 1 mm). **b**, **c** (top panels), Bright field optical images of the adjacent, non-stained section collected during the force volume AFM experiment for the CE component (**b**, top panel) and for the CL component (**c**, top panel). These areas are highlighted in (**a**). Scale bar: 100 μm. **b**, **c** (bottom panels), Elastic modulus maps (size: 60x60 μm^2, 10x10 pixels). Their approximate position is marked in blue in **b** and in orange in **c** (top panels). **d**, Elastic modulus values for patient #10, with overlapped average values and SD (2 data points are outside the axis limits).

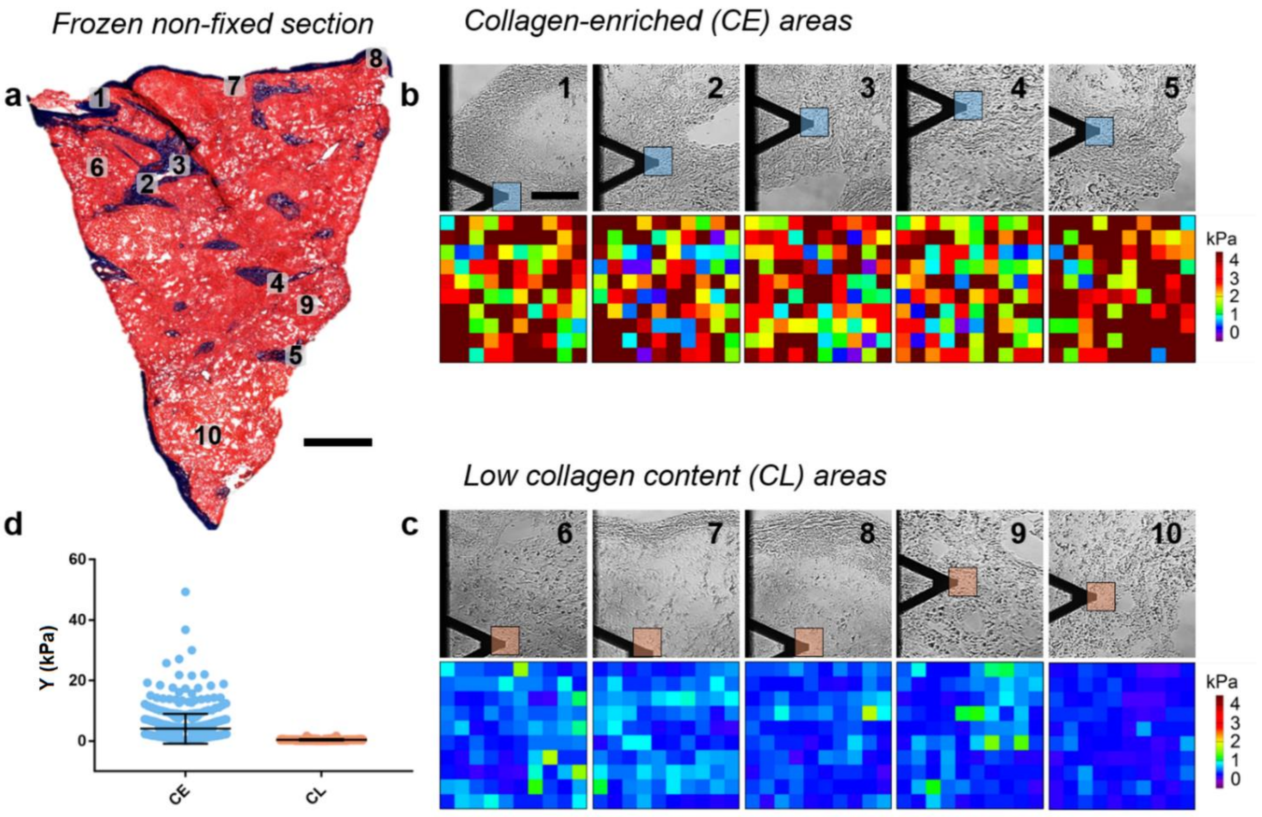

Figure S4. Force volume AFM measurements in frozen non-fixed sections from human liver. a, Reference trichrome-stained section from patient #5 (scale bar: 1 mm). **b**, **c** (top panels), Bright field optical images of the adjacent, non-stained section collected during the force volume AFM experiment for the CE component (**b**, top panel) and for the CL component (**c**, top panel). These areas are highlighted in (**a**). Scale bar: 100 μm. **b**, **c** (bottom panels), Elastic modulus maps (size: 60x60 μm^2, 10x10 pixels). Their approximate position is marked in blue in **b** and in orange in **c** (top panels). **d**, Elastic modulus values for patient #5, with overlapped average values and SD.

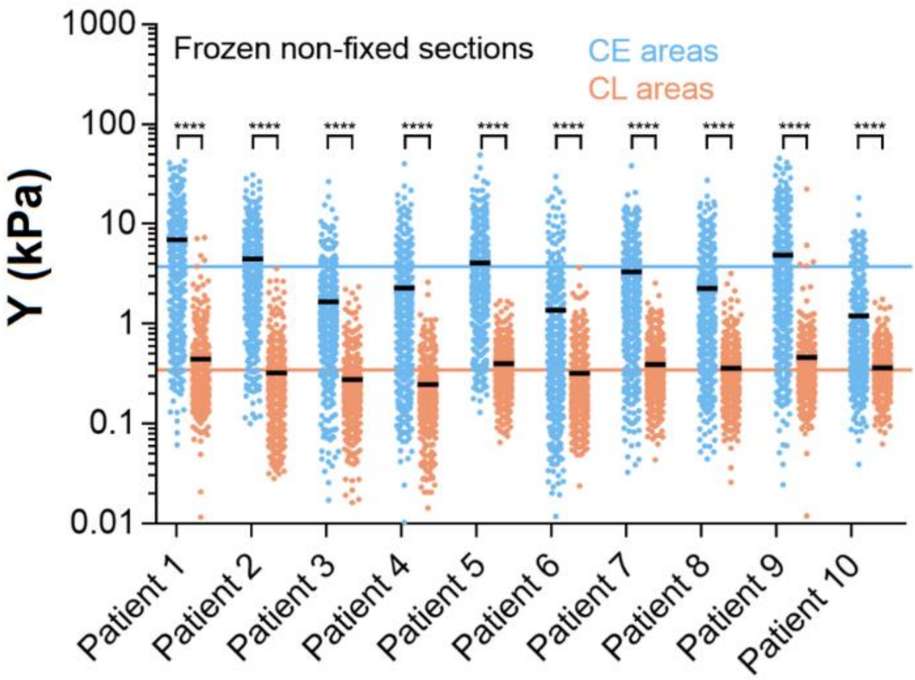

Figure S5. Statistical analysis of frozen non-fixed sections from human liver. Elastic modulus values for patients #1-10. For better visualization, data are plotted in logarithmic scale (3 data points are outside the y-axis limits), with mean values indicated in black. Pooled means with uniform samples are also shown as solid lines, separately for collagen-enriched (3.89 kPa, in blue) and low-collagen areas (0.34 kPa, in orange). For all tested patients, CE areas exhibit significantly higher Young's modulus than CL areas ($P < 0.0001$).

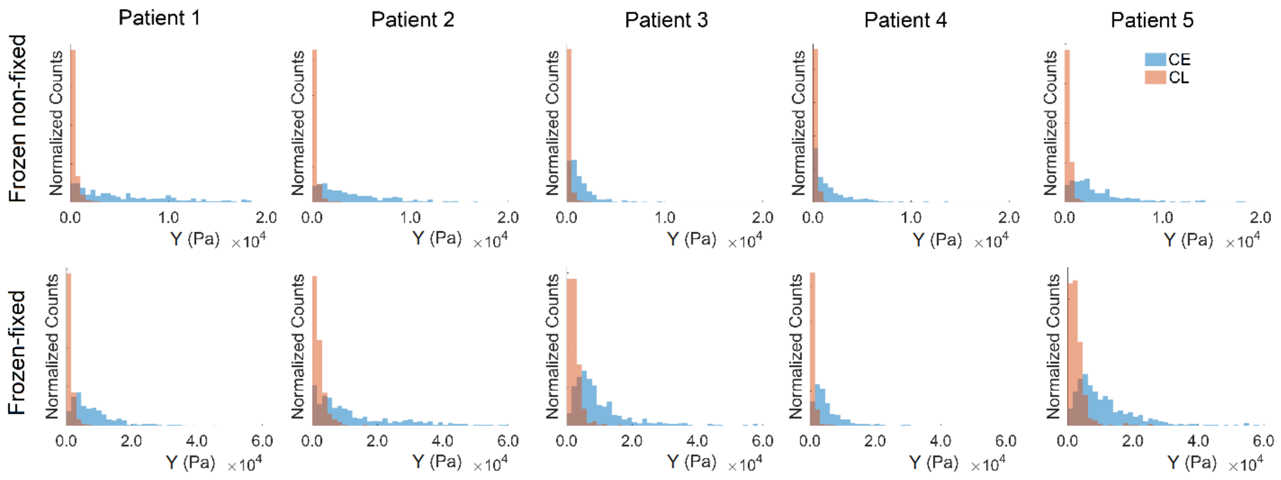

Figure S6. **Elastic modulus distribution histograms for patients #1-5.** Distribution histograms of the elastic modulus values in frozen non-fixed sections (top row) and in frozen-fixed sections (bottom row). Data are obtained from all maps (5 CE locations and 5 CL locations per patient, 10x10 points/map) collected during force volume AFM experiments. CE data are in blue, while CL data are in orange. For better visualization, some data points, i.e. 33 (patient #1), 7 (patient #2), 1 (patient #3), 3 (patient #4) and 8 (patient #5) in the CE histograms of frozen non-fixed sections, 35 (patient #2), 6 (patient #3), 1 (patient #4) and 2 (patient #5) in the CE histograms of frozen-fixed sections, are out of the x-axis limits. In frozen non-fixed sections, some CE histograms show a peak lying at higher elastic modulus values compared to the peak of the CL distribution (see data from patient #3, #4 and #5). All CE histograms are skewed to the right compared to the CL histograms. In frozen-fixed sections, some CE histograms show a distinct peak lying at higher elastic modulus values compared to the peak of the CL distribution (see data from patient #1, #3, #4 and #5). All CE histograms are skewed to the right.

Results on fixed-frozen and paraffin sections

In fixed-frozen section, the Young‘s modulus of CE and CL regions was not significantly different ($P > 0.05$), while in paraffin sections, elastic modulus was significantly higher in CL regions compared to CE regions ($P < 0.05$) (see Fig. 2 of the main text and the histograms in Fig. S7). For both preparation methods, CE and CL data were compared within each patient (see Fig. S8). Due to the long fixation time in PFA, both paraffin and fixed-frozen sections showed the highest elastic modulus among all preparation methods (pooled mean among all patients with uniform samples was 126.2 kPa for CE and 120.1 kPa for CL in fixed-frozen sections, 222.2 kPa for CE and 286.3 kPa for CL in paraffin sections, see Fig. 2 of the main text). Results indicate that, if paraffin wax is used as embedding medium, the mechanical properties of the tissues are heavily altered, as confirmed by the highest Young’s modulus. Even without paraffin embedding, overnight fixation of the whole tissue block with 4% PFA alone is enough to obscure the micromechanical heterogeneities across CE and CL regions, as observed from the results of fixed-frozen sections. The impossibility to identify collagen by the higher elastic modulus after testing sections from 5 patients justified the choice of a smaller sample size in the cases of paraffin and fixed-frozen sections.

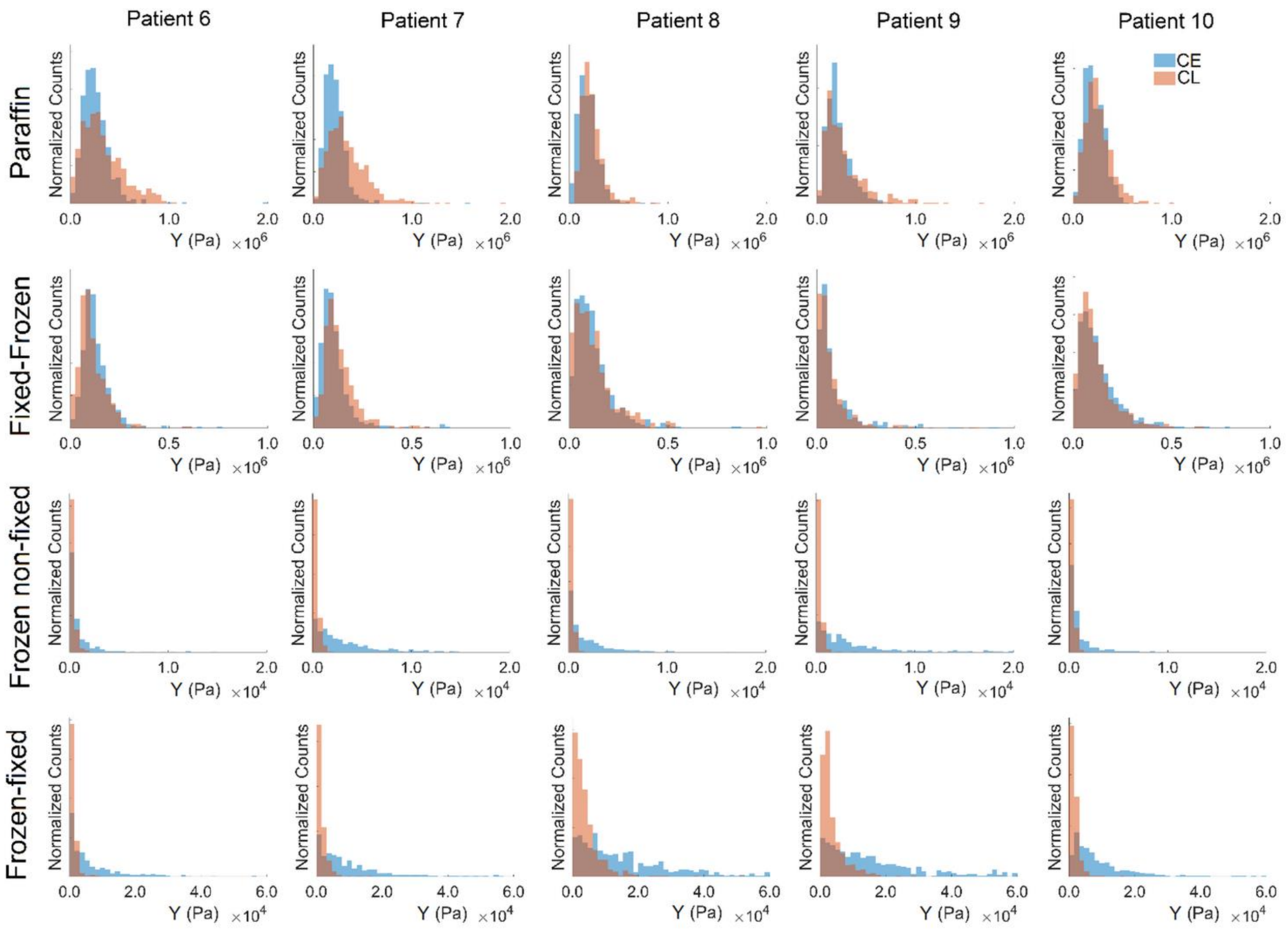

Figure S7. **Elastic modulus distribution histograms for patients #6-10.** Distribution histograms of the elastic modulus values in paraffin sections (first row from top), fixed-frozen sections (second row from top), frozen non-fixed sections (third row from top) and frozen-fixed sections (bottom row). Data are from all force maps (5 CE/5 CL areas/patient, 10x10 points/map), collected during force volume AFM experiments. For better visualization, some data points, i.e. 1 (patient #7), 1 (patient #9) in the CE histograms of paraffin sections, 1 (patient #6), 1 (patient #8), 2 (patient #9), 3 (patient #10) in the CE histograms of fixed-frozen sections, 4 (patient #8), 2 (patient #10) in the CL histograms of fixed-frozen sections, 3 (patient #6), 2 (patient #7), 5 (patient #8), 25 (patient #9) in the CE histograms of frozen non-fixed sections, 5 (patient #9) in the CL histogram of frozen non-fixed sections, 3 (patient #6), 4 (patient #7), 26 (patient #8), 17 (patient #9), 6 (patient #10) in the CE histograms of frozen-fixed sections, 1 (patient #9), 1 (patient #10) in the CL histograms of frozen-

fixed sections, are out of the x-axis limits. In frozen non-fixed sections, CE histograms from patients #6-10 are skewed to the right compared to the histograms from CL areas. In frozen-fixed sections, the CE histogram from patient #10 shows a distinct peak at higher elastic modulus values. All CE histograms are skewed to the right compared to the corresponding histograms from CL areas.

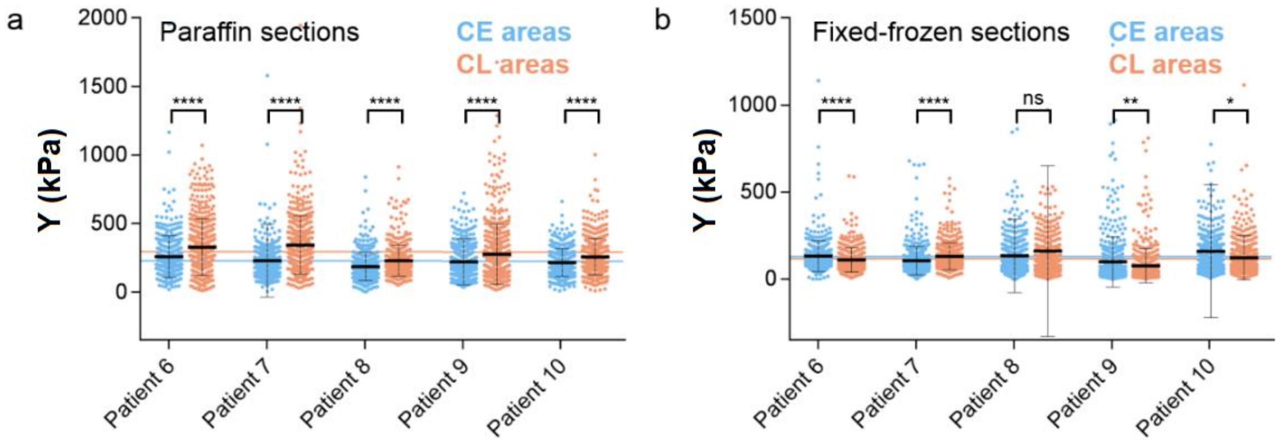

Figure S8. Statistical analysis of paraffin and fixed-frozen sections from human liver. a, **b**, Elastic modulus values for patients #6-10 in paraffin (**a**) and in fixed-frozen (**b**) sections, with overlapped average values (in black). In paraffin sections, CL areas exhibit significantly higher Young's modulus than CE areas for all tested patients individually ($P < 0.0001$). In fixed-frozen sections, we found inconsistent results among patients. $Y_{CE\ areas}$ was significantly higher than $Y_{CL\ areas}$ in patient #6 ($P < 0.0001$), patient #9 ($P < 0.01$) and patient #10 ($P < 0.05$). CE and CL values were not statistically different for patient #8 and $Y_{CL\ areas}$ was significantly higher than $Y_{CE\ areas}$ for patient #7 ($P < 0.0001$). For better visualization, 2 data points (**a**) and 9 data points (**b**) are outside the y-axis limits. Pooled means among patients with uniform samples are also shown as blue and orange lines.

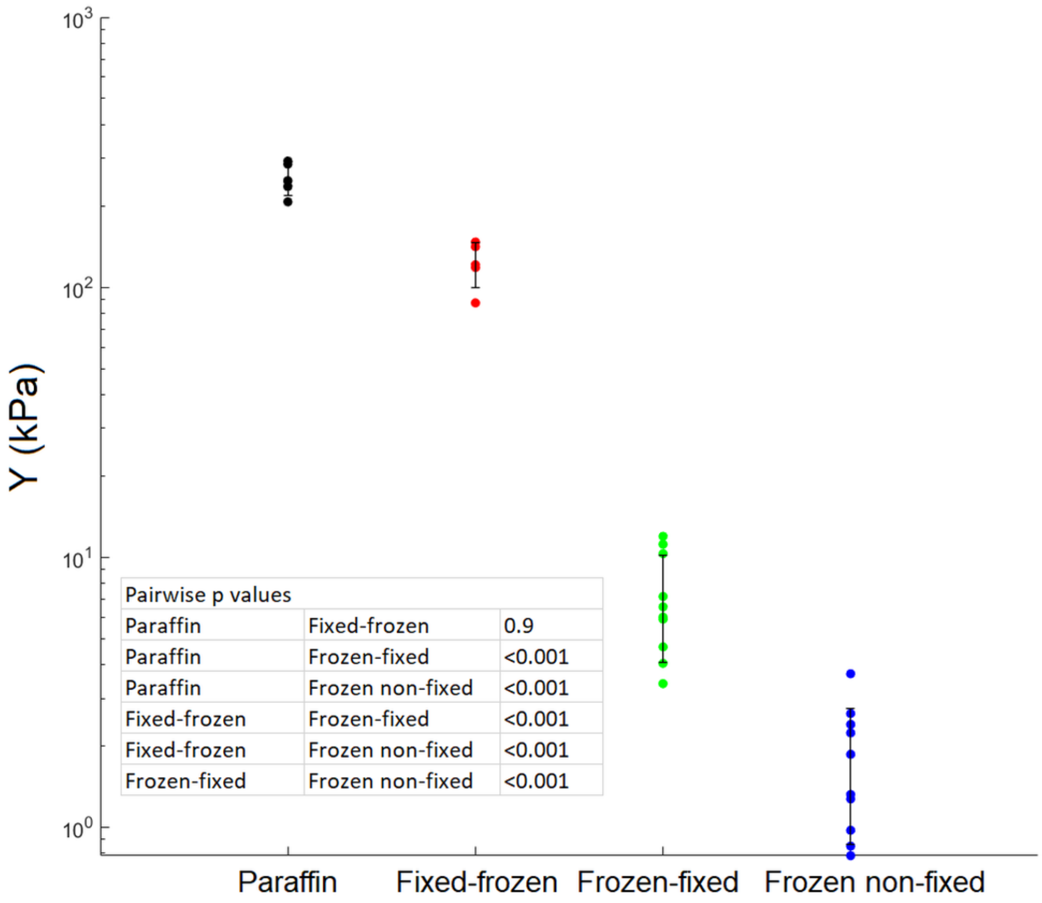

Figure S9. Effect of the preparation method on the elastic modulus of tissue sections. Average from each sample (combining CE+CL areas) for each method. To compare the overall elastic modulus among preparation conditions, all elastic modulus values for CE and CL regions are combined per patient. A global one-way ANOVA showed significant difference (p=1.4x10-20) (Matlab). ANOVA was followed by Tukey-Kramer multicomparison testing using default parameters (Matlab). Means and standard deviations are plotted with elastic modulus data in log scale with pairwise p values indicated in the table. Only the couple paraffin-fixed frozen samples exhibited no statistical difference ($p > 0.9$).

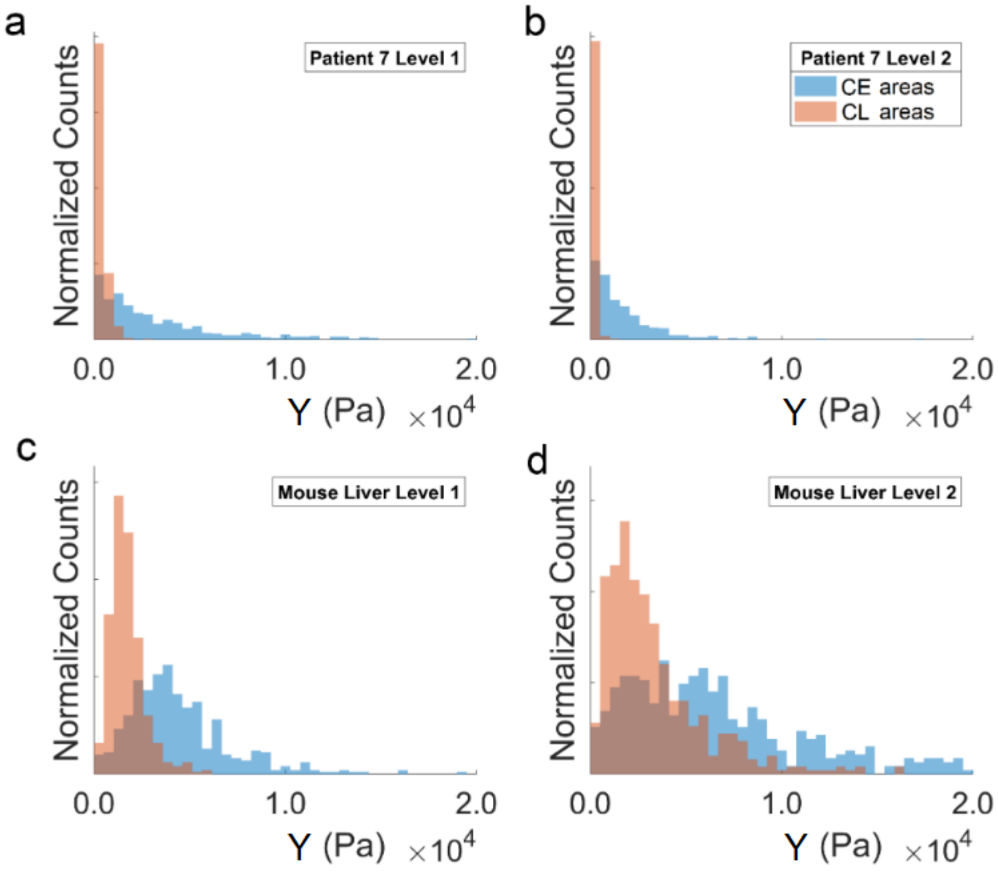

Figure S10. Elastic modulus measurements of different sections within the same tissue block, in frozen non-fixed human sections and in frozen-fixed mouse sections. a, **b,** Elastic modulus distribution histograms of two frozen non-fixed sections from human liver belonging to the same patient (patient #7), here called levels. Distance between levels is 200 µm. **c**, **d,** Elastic modulus distribution histograms of two frozen-fixed sections from mouse liver, which are 200 µm far apart. For better data visualization 2 data points in the CE histogram in (**a**) and 6 data points in the CE histogram in (**d**) are out of the x-axis limits.

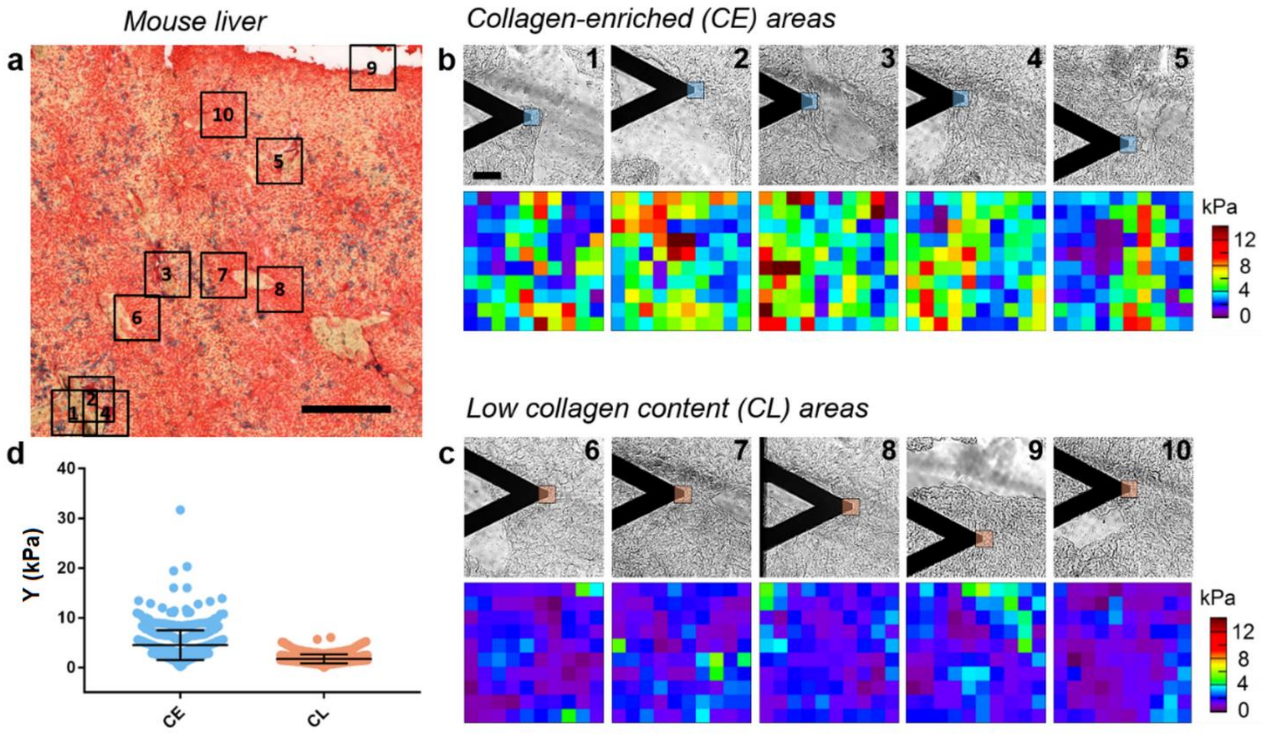

Figure S11. Force volume AFM measurements in frozen-fixed sections from mouse liver. a, Reference trichrome-stained section (scale bar: 500 μm). **b, c** (top panels), Bright field optical images from the adjacent section collected during the force volume experiment, corresponding to regions of interest for the CE component (**b**, top panel) and for the CL component (**c**, top panel). These areas are highlighted in (**a**). Scale bar: 50 μm. **b, c** (bottom panels), Elastic modulus maps (size: 30x30 μm^2, 10x10 pixels). Their approximate position is marked in blue in **b** (top panel) and in orange in **c** (top panel). **d**, Elastic modulus values for mouse liver, obtained from all the CE and CL maps (5CE/5CL areas, 10x10 points/area) with overlapped average values and SD.

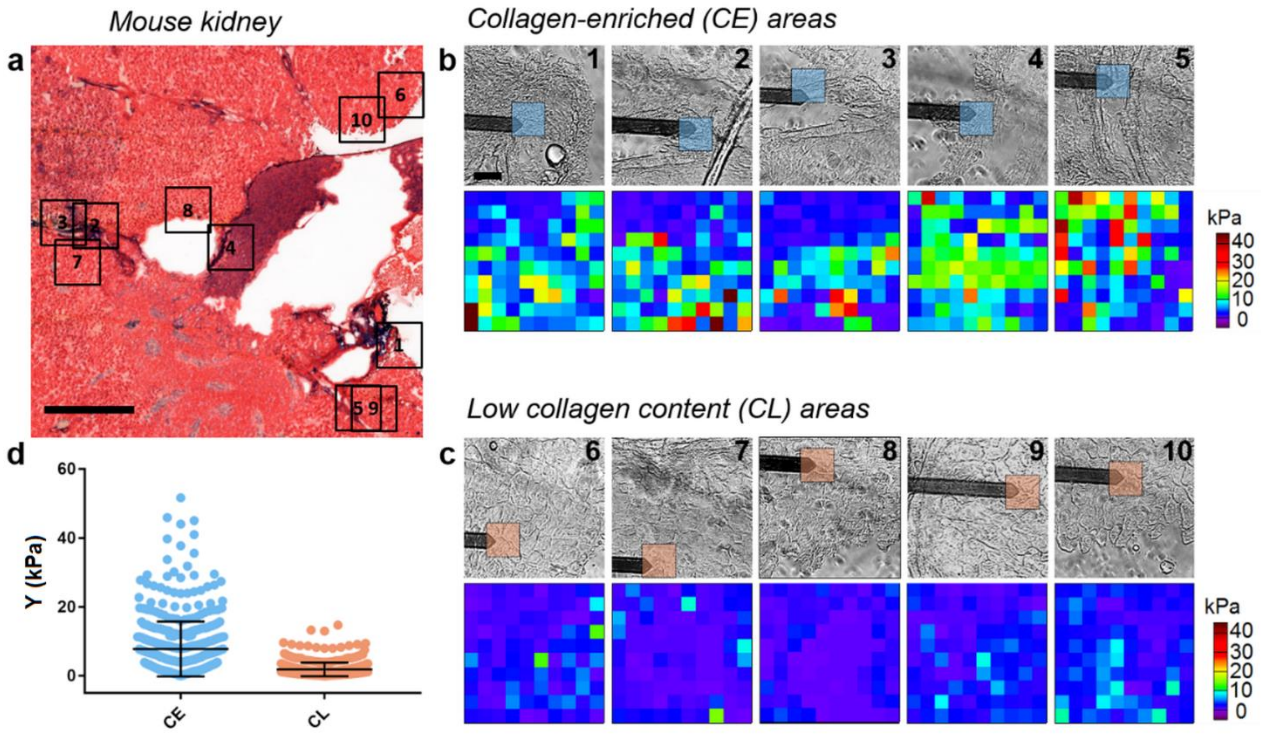

Figure S12. Force volume AFM measurements in frozen-fixed sections from mouse kidney. a, Reference trichrome-stained section (scale bar: 500 μm). **b**, **c** (top panels), Bright field optical images from adjacent section, collected during the force volume experiment for the CE component (**b**, top panel) and for the CL component (**c**, top panel). These areas are highlighted in (**a**). Scale bar: 50 μm. **b**, **c** (bottom panels), Elastic modulus maps (size: 60x60 μm^2, 10x10 pixels). Their approximate position is marked in blue in **b** (top panel) and in orange in **c** (top panel). **d**, Elastic modulus values for mouse kidney, with overlapped average values and SD.

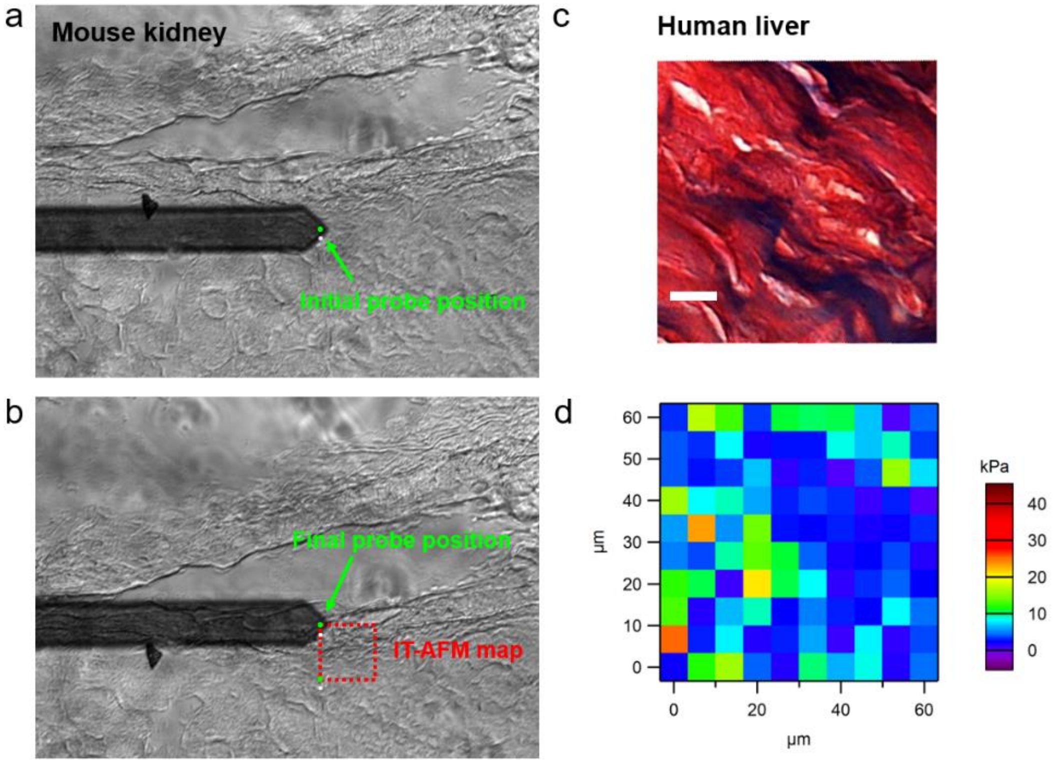

Figure S13. Localization of the force map in a tissue section from mouse kidney and spatial correlations between a trichrome ROI and a elastic modulus map from human liver. a, **b**, Bright field optical image of a section from mouse kidney taken at the beginning (**a**) and at the end (**b**) of the force volume mapping. The position of the probe (visible under the AFM optics) is highlighted in green, while the area of the map, highlighted in red, is 50x50 μm^2 (see also Fig. 5 of the manuscript). **c**, **d,** Region of interest (ROI) in a trichrome-stained section from human liver (scale bar: 10 µm) (**c**) and corresponding 60x60 μm^2 elastic modulus map in the non-stained section (**d**). Data belong to patient #1.

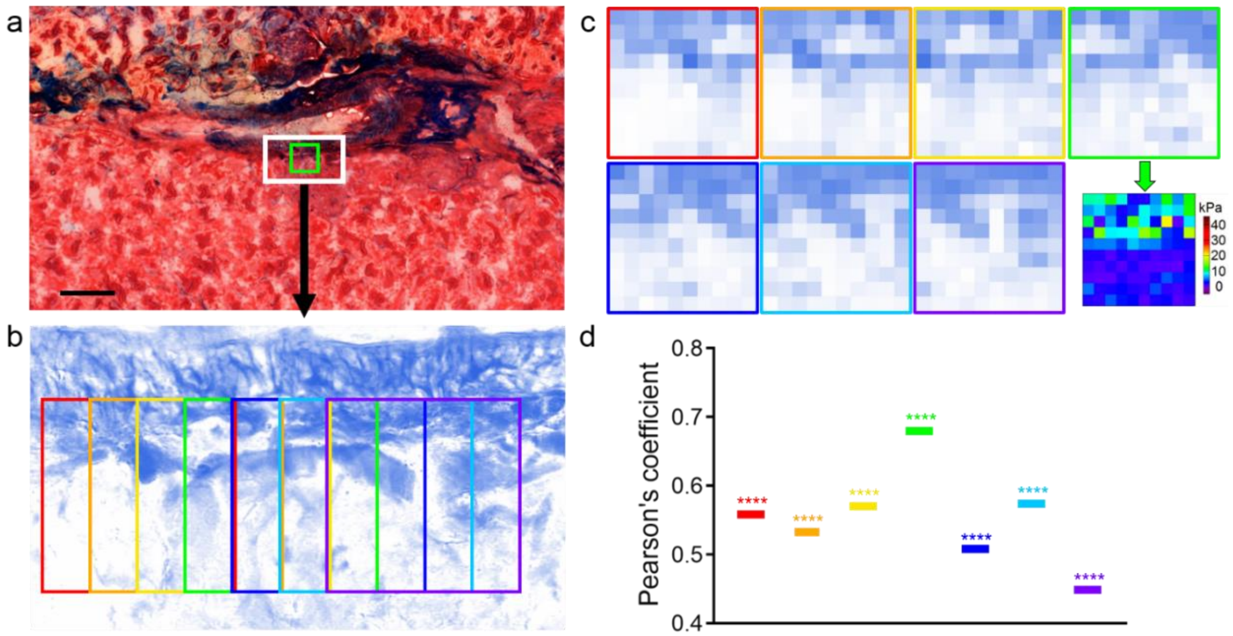

Figure S14. Spatial correlation analysis. a, Trichrome-stained reference section showing a collagen-enriched area around a blood vessel in a frozen-fixed tissue section from mouse kidney (scale bar: 100 µm). **b**, Color deconvoluted rectangular area of the section (size: 150x80 µm^2), marked in white in (**a**), where ROIs of size 50x50 µm^2 were sectioned out for correlation analysis (see also the Methods Section). **c**, Tiled ROIs at the CE-CL interface spaced by 6.1 µm along the horizontal direction. **d**, Results of the correlation analysis, i.e. Pearson's coefficients as extracted by comparing pixel by pixel the ROIs in (**c**) with the low-resolution (10x10 pixels) elastic modulus map collected during the force volume AFM experiment. The low-resolution elastic modulus map is shown in (**c**), bottom image on the right. Obtained Pearson's coefficients were not affected by slightly rotating the rectangular area in (**a**) and (**b**) by ± 5 degrees.

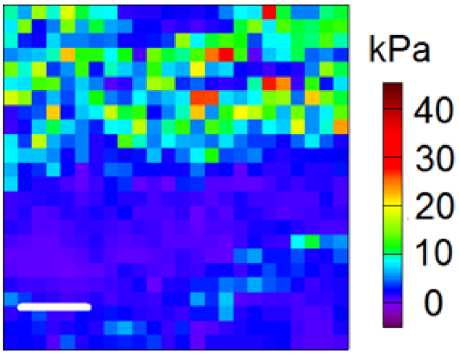

Figure S15. High resolution Young's modulus map from mouse kidney. The force volume map (24x24 pixels, 50x50 μm^2) is collected in the same sample region as the one highlighted in blue in Fig. 5d (inset) of the manuscript (acquisition time: 10 minutes).

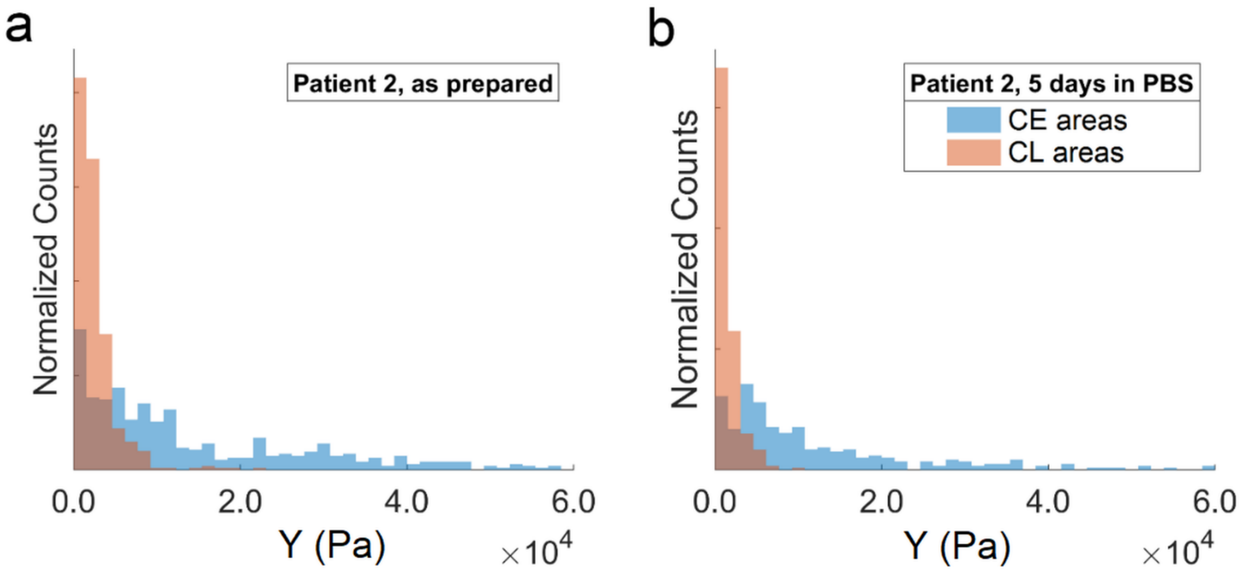

Figure S16. Time-stability of frozen-fixed tissue sections. a, **b,** Elastic modulus distribution histograms of a frozen-fixed section immediately after preparation (**a**) and after 5 days of storage in PBS at 4°C (**b**). Data belong to patient #2. For better data visualization, 31 data points for CE distribution (**a**) and 4 data point for CE distribution (**b**) are out of the x-axis limits.

Pairwise Significance (p) of Patient Difference CE Regions Via Multi-comparison Procedure

	1	2	3	4	5	6	7	8	9	10
1		> 0.05	> 0.05	< 0.05	> 0.05	< 0.001	< 0.001	< 0.001	< 0.001	< 0.001
2			> 0.05	> 0.05	< 0.001	< 0.001	< 0.001	< 0.001	< 0.001	< 0.001
3				< 0.05	> 0.05	< 0.001	< 0.001	< 0.001	< 0.001	< 0.001
4					< 0.001	< 0.001	< 0.001	< 0.001	< 0.001	< 0.001
5						< 0.001	< 0.001	< 0.001	< 0.001	< 0.001
6							> 0.05	> 0.05	> 0.05	> 0.05
7								> 0.05	> 0.05	> 0.05
8									> 0.05	> 0.05
9										> 0.05
10										

Pairwise Significance (p) of Patient Difference CL Regions Via Multi-comparison Procedure

	1	2	3	4	5	6	7	8	9	10
1										
2	> 0.05									
3	< 0.001	> 0.05								
4	< 0.05	< 0.001	< 0.001							
5	> 0.05	> 0.05	< 0.05	< 0.001						
6	< 0.001	< 0.001	< 0.001	< 0.001	< 0.001					
7	< 0.001	< 0.001	< 0.001	< 0.001	< 0.001	> 0.05				
8	< 0.001	< 0.001	< 0.001	< 0.001	< 0.001	> 0.05	> 0.05			
9	< 0.001	< 0.001	< 0.001	< 0.001	< 0.001	> 0.05	> 0.05	> 0.05		
10	< 0.001	< 0.001	< 0.001	< 0.001	< 0.001	> 0.05	> 0.05	> 0.05	> 0.05	

Table S1. Pairwise patients differences via multi-comparison procedure. To compare patient elasticity values, averages for all collagen and non-collagen regions were separately computed for each patient, compared by ANOVA and multiple comparison methods. A global one-way ANOVA performed in MATLAB showed significant difference (p=1.4x10-20). ANOVA was followed by Tukey-Kramer multicomparison testing in MATLAB using default parameters. The tables show pairwise p values in blue (CE) and orange (CL).

Sample	# Sections measured by AFM	# CE maps/section	# points/map	Map's size
Patient 1	2*	5 CE/5 CL	100	60x60μm^2
Patient 2	2*	5 CE/5 CL	100	60x60μm^2
Patient 3	2*	5 CE/5 CL	100	60x60μm^2
Patient 4	2*	5 CE/5 CL	100	60x60μm^2
Patient 5	2*	5 CE/5 CL	100	60x60μm^2
Patient 6	4**	5 CE/5 CL	100	60x60μm^2
Patient 7	4** + 1***	5 CE/5 CL	100	60x60μm^2
Patient 8	4**	5 CE/5 CL	100	60x60μm^2
Patient 9	4**	5 CE/5 CL	100	60x60μm^2
Patient 10	4**	5 CE/5 CL	100	60x60μm^2
Mouse liver	2****	5 CE/5 CL	100	30x30μm^2
Mouse kidney	1*****	5 CE/5 CL<br>1 (CE/CL interface)******<br>1 (CE/CL interface)******	100<br>100<br>576	60x60μm^2<br>50x50μm^2<br>50x50μm^2

* one frozen-fixed section, one frozen non-fixed section
** one section for each preparation method
*** frozen non-fixed section spaced by 200 μm from the first frozen non-fixed section measured
**** frozen-fixed sections spaced by 200 μm from each other
***** frozen-fixed section
****** maps of the same area

Table S2. Statistical dataset.
